# Supertertiary protein structure affects an allosteric network

**DOI:** 10.1073/pnas.2007201117

**Published:** 2020-09-14

**Authors:** Louise Laursen, Johanna Kliche, Stefano Gianni, Per Jemth

**Affiliations:** ^a^Department of Medical Biochemistry and Microbiology, Uppsala University, SE-75123 Uppsala, Sweden;; ^b^Istituto Pasteur-Fondazione Cenci Bolognetti, Dipartimento di Scienze Biochimiche “A. Rossi Fanelli,” Sapienza Università di Roma, 00185 Roma, Italy;; ^c^Istituto di Biologia e Patologia Molecolari, Consiglio Nazionale delle Ricerche, Sapienza Università di Roma, 00185 Roma, Italy

**Keywords:** allostery, protein interactions, double-mutant cycle, kinetics, supertertiary structure

## Abstract

The notion that protein function is allosterically regulated by structural or dynamic changes in proteins has been extensively investigated in several protein domains in isolation. In particular, PDZ domains have represented a paradigm for these studies, despite providing conflicting results. Furthermore, it is still unknown how the association between protein domains in supramodules, consitituting so-called supertertiary structures, affects allosteric networks. Here, we experimentally mapped the allosteric network in a PDZ:ligand complex, both in isolation and in the context of a supramodular structure, and show that allosteric networks in a PDZ domain are highly dependent on the supertertiary structure in which they are present. This striking sensitivity of allosteric networks to the presence of adjacent protein domains is likely a common property of supertertiary structures in proteins. Our findings have general implications for prediction of allosteric networks from primary and tertiary structures and for quantitative descriptions of allostery.

## Significance

Protein function can be allosterically regulated by changes in structure or dynamics. PDZ domains are classic examples for studies of allostery in single protein domains. However, PDZ domains are often found in multidomain proteins; in particular, PDZ3 is located in a supramodule containing three domains. The allosteric network in PDZ3 has never been studied in the presence of the adjacent domains. Here we map the allosteric network for a PDZ3:ligand complex, both in isolation and in the context of a supramodule. We demonstrate that the allosteric network is highly dependent on this supertertiary structure, with broad implications for studies of allostery in single domains.

Allosteric regulation is an essential function of many proteins, well established in multimeric proteins with well-defined conformational changes. From a biological perspective, such allostery plays important roles, for example in regulation of enzyme activity and binding of oxygen to hemoglobin, which was the basis for models such as MWC (1) and KNF (2). More recently the concept has been extended to monomeric proteins (3) and intrinsically disordered proteins (4, 5). Here, allostery is a process where a signal propagates from one site to a physically distinct site, although the exact mechanism is elusive (6). Thus, as experimental approaches have developed the definition of the allosteric mechanism has evolved too and it is now spanning from the classical structure-based allostery to the ensemble nature of allostery and provides the framework for capturing allostery from rigid and structured proteins to dynamic and disordered proteins (7).

**Fig. 1.**
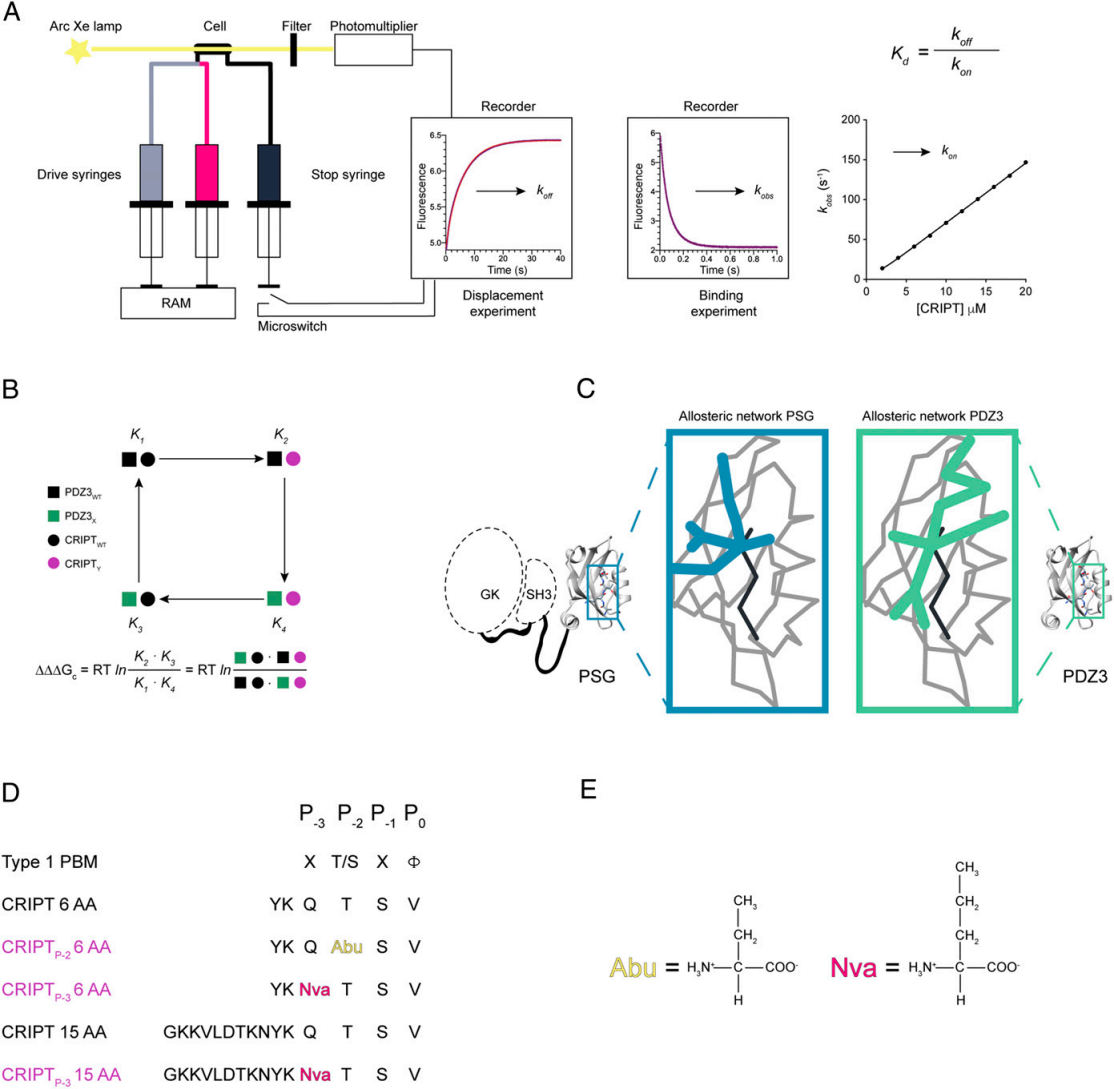
Illustration of experimental setup. (*A*) Setup for the stopped-flow experiments and kinetic traces from displacement and binding experiments. *k*_obs_ values from binding experiments were plotted versus peptide concentration to obtain the association rate constant (*k*_on_), whereas the dissociation rate constant (*k*_off_) was obtained from displacement experiments. From these kinetic parameters, *K*_d_ values were calculated for each protein:peptide complex. (*B*) Illustration of a double-mutant cycle used to obtain the coupling free energy (ΔΔΔG_c_) between a residue in PDZ3 and in the CRIPT peptide ligand. (*C*) Illustration of the overall question, to explore if the allosteric network in a single domain is dependent on the presence of supertertiary structure. To address the question, we compared the allosteric network within PDZ3 in the presence (blue) and absence (green) of the SH3 and GK domains. (*D*) The different CRIPT peptides used in our experiments. Color codes: black represents WT CRIPT 6 AA or 15 AA and purple is CRIPT with a single mutation. A type 1 PBM is defined by a hydrophobic residue at P_0_ and a Thr or Ser at P_-2_. The residues with mutated side chains are highlighted: P_-2_(Abu) and P_-3_(Nva). (*E*) Side chains for Abu (2-amino-butyric acid) and Nva (norvaline).

PDZ3 from PSD-95 has been extensively used as a model system for allostery in a single protein domain. Originally, it was used as a benchmark of a statistical method to predict allostery from covariation of mutations (8) and has since then been subject to study by numerous methods (9). PDZ domains are small protein domains, around 100 amino acid residues, with a specific fold that contains five or six β strands and two or three α-helices (Fig. 1*C*). The PDZ domain family is one of the most abundant protein–protein interaction domains in humans and it is often found in scaffolding or signaling proteins. PDZ domains bind to short interaction motifs (three to six residues), usually at the C terminus of ligand proteins. Looking at PDZ3 as a system to understand principles that underlie allosteric regulation reveals the complexity of the phenomenon. The first allosteric network in PDZ3 was determined by statistical coupling analysis and depicted a network that propagates from the binding pocket (α_2_ helix, β_2_ sheet, and β_1_β_2_ loop) to the proposed allosteric site around the β_1_ and β_2_ strand. A part of the allosteric network was recently experimentally validated by a deep coupling scan (10) for the α_2_ helix in the PDZ3. However, their analysis was only applied to residues in direct contact with the peptide ligand. In fact, development of new approaches to capture allosteric networks in proteins have produced several distinct but sometimes overlapping allosteric networks in PDZ3. Intriguingly, no two identical networks have been reported. The overall disagreement suggests that the resulting allosteric network is influenced by the choice of method to map it (9). A likely reason for the discrepancy is that allostery is caused by small energetic internal redistributions upon perturbation (11) in an overall cooperatively folded protein domain. It is clear that the context of a protein domain matters for its stability (12). Clearly, if the networks are so sensitive, it appears likely that the structural context of the protein domain would also affect any allosteric network.

Most PDZ domains are part of multidomain proteins. For example, PDZ3 is part of PSD-95, a scaffolding protein from the postsynaptic density that contains three PDZ domains and one SH3 and one guanylate kinase (GK) domain. PDZ1 and PDZ2 form a tandem domain supramodule, whereas PDZ3, SH3, and GK form another supramodule, denoted PSG (Fig. 1*C*), which is found in the whole MAGUK protein family to which PSD-95 belongs. Function and binding have been shown to be dependent on the interdomain architecture and dynamics, that is, the supertertiary structure of the supramodules PDZ1-2 and PSG (13–15). Notably, while the allosteric networks for PDZ3 have been extensively studied, they have not been assessed in the context of the PSG supramodule. A few studies report a contribution to the allosteric network in PDZ3 from the α_3_ helix which connects PDZ3 with the SH3–GK tandem (16, 17) (*SI Appendix*, Fig. S1). The potential role of the adjacent domains in the supramodule is particularly relevant since binding of protein ligands to the SH3–GK tandem is modulated by binding of cysteine-rich interactor of PDZ3 (CRIPT) to PDZ3 (18).

To address the question of structural context for allosteric networks in single protein domains we mapped coupling free energies (ΔΔΔG_c_) upon peptide ligand binding to PDZ3 and PSG, respectively, using the double-mutant cycle approach (Fig. 1*B*) (19). A direct comparison of the mapped allosteric networks showed that similar patterns of energetic coupling were present around the binding pocket in both isolated PDZ3 and the PSG. However, the presence of the SH3–GK tandem resulted in strong coupling from the bound peptide ligand to the β_1_β_2_ loop, β_2_β_3_ loop, and α_3_ helix that was not observed with the single PDZ3 domain. Our results show that the allosteric energy landscape of PDZ3 is highly sensitive to supertertiary structure, in agreement with the conflicting previous studies on isolated PDZ domains.

## Results

We set out to test whether the allosteric network in PDZ3 is dependent on the supertertiary structure in which it is present, that is, the interdomain organization and dynamics of the PSG supramodule (Fig. 1*C*). To address this question and seek general principles that determine allosteric signal propagation in small protein domains we measured and compared coupling free energies (ΔΔΔG_c_) from mutational perturbations for PDZ3 and PSG, respectively. We also asked the following two questions for both PDZ3 and PSG: How is the allosteric network, as defined by the pattern of ΔΔΔG_c_ values, affected by the origin of perturbation (i.e., the residue, which is mutated in the peptide), and how is the allosteric network affected by the length of the peptide ligand? We mapped the allosteric network using N-terminally dansyl-labeled peptides derived from the ligand CRIPT (with either the last 6 amino acids or the last 15 amino acids, respectively, from CRIPT) with mutations at either position P_-2_ or P_-3_ (Fig. 1 *D* and *E*). The peptides used were CRIPT 6 AA (YKQTSV), CRIPT_P-3_ 6 AA (YKNvaTSV), CRIPT_P-2_ 6 AA (YKQAbuSV), CRIPT 15 AA (GKKVLDTKNYKQTSV), and CRIPT_P-3_ 15 AA (GKKVLDTKNYKNvaTSV). We could not obtain data of high enough quality for a quantitative analysis with CRIPT_P-2_ 15 AA (GKKVLDTKNYKQAbuSV).

### Experimental Setup: Double-Mutant Cycles as a Method to Map Allosteric Networks.

In this study we applied double-mutant cycles to quantify the intermolecular interaction between residues in PDZ3 and residues in CRIPT expressed as a coupling free energy, ΔΔΔG_c_ (Fig. 1*B*). Double-mutant cycle is a powerful approach (20, 21) and it has been applied to both intra- and intermolecular protein interactions (22–25). Our experimental setup was to measure kinetic rate constants for association (*k*_on_) and dissociation (*k*_off_) by stopped-flow experiments to deduce the affinity for the PDZ3:CRIPT interaction from the ratio *k*_off_/*k*_on_ (Fig. 1*A*). Such rate constants have a high accuracy and precision, which results in relatively small errors in ΔΔΔG_c_. One experimental error is in the concentration of ligand, which directly affects *k*_on_, but the dansyl group used in the experiments provides accurate determination by absorbance. The concentration of protein does not affect *k*_on_ since peptide is in excess. *k*_off_ is a first-order rate constant and in a displacement experiment it is not sensitive to errors in either peptide or protein concentration.

The study was performed using our previously developed experimental system in which a Trp residue was engineered into PDZ3 (F337W) to enhance the change in fluorescence upon peptide ligand binding. F337W was previously shown not to affect the affinity of PDZ3 for the CRIPT interaction (26). In the present study we recorded circular dichroism (CD) spectra (200 to 260 nm) and determined the affinity by isothermal titration calorimetry (ITC) for PDZ3 and PSG with and without the Trp337 probe (*SI Appendix*, Fig. S2). The data showed that the F337W probe did not affect secondary structure or affinity and the pseudo wild types (containing F337W) are therefore referred to as WT PDZ3 and WT PSG.

In a double-mutant cycle WT PDZ3, mutant PDZ3, WT CRIPT, and mutant CRIPT were used to obtain four sets of *k*_*on*_ and *k*_*off*_ values for each coupling free energy. Thus, from the rate constants the affinities (expressed as dissociation constants, *K*_d_ values) of the following four complexes were calculated: 1) PDZ3_WT_:CRIPT_WT_, 2) PDZ3_X_:CRIPT_WT_, 3) PDZ3_WT_:CRIPT_Y_, and 4) PDZ3_X_:CRIPT_Y_, where X and Y denote the respective mutation in PDZ3 and CRIPT (Fig. 1*B*). The *K*_d_ values can be used to calculate ΔΔΔG_c_ between residue X in PDZ3 and residue Y in CRIPT. If the product of binding constants for single mutants equals the product of binding constants for WT and double mutant, then ΔΔΔG_c_ = 0 and no intermolecular interaction energy (or “communication”) exists between the probed residues X and Y. However, if ΔΔΔG_c_ ≠ 0 an intermolecular interaction is present between the two residues. We included a total of 32 mutations in PDZ3, 15 mutations in PSG, 2 mutations in CRIPT 6 AA, and 1 mutation in CRIPT 15 AA, respectively. An example of a calculation is shown in *SI Appendix*, Fig. S3. All calculations are provided in Dataset S1 *A*–*D*.

In general we used conservative deletion mutations to conform to the assumptions underlying interpretation of mutations in proteins (27). Hence, residues in PDZ3 and PSG were substituted with Ala to prevent new interactions or steric clashes to be formed, the only exceptions being Ile→Val (also a conservative deletion mutation) and Gly→Ala. CD spectra were recorded for all mutants to ensure intact secondary structure (*SI Appendix*, Fig. S4). Binding kinetics (*k*_*on*_, *k*_*off*_) were studied for each mutant with all five peptide ligands included, that is, CRIPT 6 AA, CRIPT_P-3_ 6 AA, CRIPT_P-2_ 6 AA, CRIPT 15 AA and CRIPT_P-3_ 15 AA (Fig. 1*D* and *SI Appendix*, Fig. S5). We chose unnatural amino acids to make the CRIPT mutations conservative, Thr→Abu and Gln→Nva, respectively. These mutations probe the effect of the functional groups on Thr (hydroxyl) and Gln (amide) without changing the length of the hydrocarbon chain (Fig. 1*E*). Finally, we want to point out that mutation, or any other experimental perturbation used to probe a protein, can potentially affect structural or indeed allosteric ground states. Therefore, a large set of mutations must be included and the overall picture assessed. In the present case, the kinetics of virtually all PSG mutants are similar, including double exponential phases and kinetic amplitudes with comparable concentration dependences.

### Patterns of energetic coupling from two peptide positions overlap in the isolated PDZ3 domain.

**Fig. 2.**
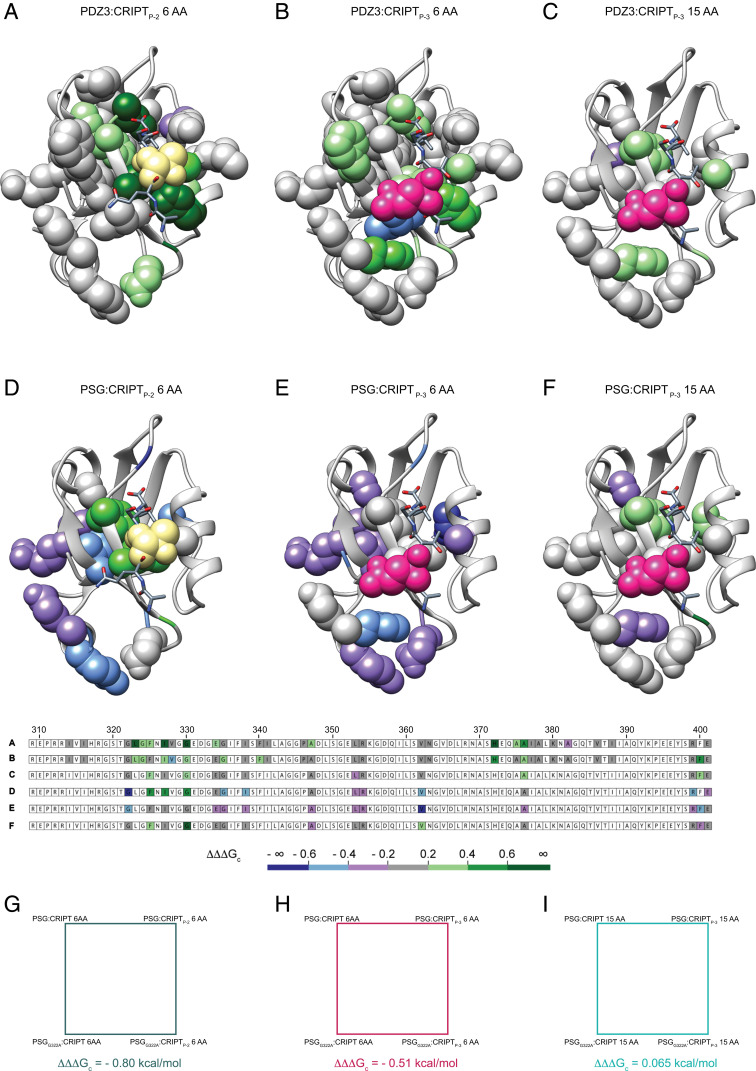
The allosteric network in PDZ3 is modulated by peptide side chain, peptide length, and presence of SH3–GK. The allosteric network, defined in terms of ∆∆∆G_c_ values mapped onto the PDZ3:CRIPT complex (Protein Data Bank ID code: 1be9) for six different cases, where *A*–*C* display data on the isolated PDZ3 domain and *D*–*F* on the PSG supramodule. (*A* and *D*) Coupling free energies between the hydroxyl group of Thr_-2_ in CRIPT to residues in single domain PDZ3 (*A*) or PDZ3 in PSG (*D*) (peptide perturbation Thr_-2_ → Abu, CRIPT_P-2_ 6 AA). (*B* and *E*) Coupling free energies from the amide moiety of Gln_-3_ in CRIPT to residues in single domain PDZ3 (*B*) or PDZ3 in PSG (*E*) (CRIPT_P-3_ 6 AA, peptide perturbation Gln_-3_ → Nva). (*C* and *F*) Coupling free energies from the amide moiety of Gln-3 in CRIPT to residues in single domain PDZ3 (*C*) or PDZ3 in PSG (*F*) (peptide perturbation Gln_-3_ → Nva, CRIPT_P-3_ 15 AA). The residue probed by mutation in CRIPT is shown as yellow (P_-2_, Thr_-2_ → Abu) or pink (P_-3_, Gln_-3_ → Nva) spheres. The side chains probed by mutation in PDZ3 are depicted as spheres with a color code corresponding to the coupling free energy between the peptide and PDZ3 side chains. The primary structure of PDZ3 (residues 309 to 401) is shown below the structures and color-coded based on coupling free energies. (*G*, *H*, and *I*) Three examples of double-mutant cycles involving the G322A mutation in PDZ3 and Thr_-2_ → Abu or Gln_-3_ → Nva in CRIPT. Each resulting coupling free energy is represented by side chains depicted as spheres in *A*–*F*.

**Fig. 3.**
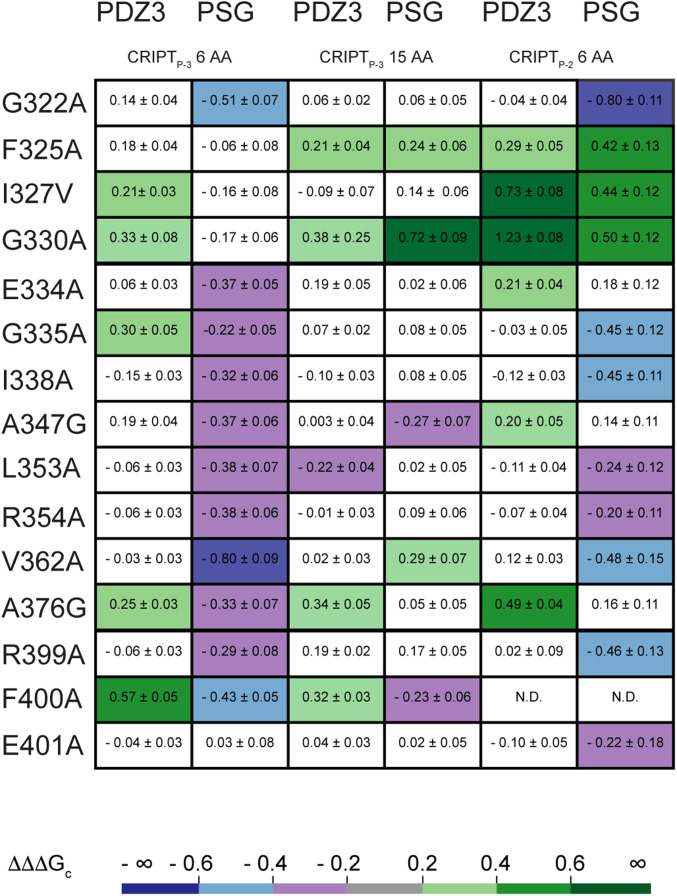
Coupling free energies (∆∆∆G_c_) for side chains in PDZ3 and PSG to side chains in CRIPT. Values of coupling free energies between 15 side chains in PDZ3 or PSG and two CRIPT side chains in either a 6-mer or a 15-mer CRIPT peptide, resulting in six allosteric networks: PDZ3:CRIPT_P-3_ 6 AA, PSG:CRIPT_P-3_ 6 AA, PDZ3:CRIPT_P-3_ 15 AA, PSG:CRIPT_P-3_ 15 AA, PDZ3:CRIPT_P-2_ 6 AA, and PSG:CRIPT_P-2_ 6 AA.

The allosteric network in PDZ3 was analyzed by mutating 32 out of 93 residues based on residue properties and previous studies. Thus, the mutations probed hydrophobic interactions (22) and charge properties (17), the role of the α_3_ helix (15), and residues important for folding (28) and for dynamics of PDZ3 (29) (Figs. 2 and 3 and *SI Appendix*, Tables S1, S2, and S3 and Dataset S1). The coupling free energy for each position was mapped onto the structure of PDZ3 to visualize the allosteric network (Fig. 2). Comparison of the coupling free energies reported by the two different peptide perturbations in CRIPT_P-2_ 6 AA (Thr→Abu) and CRIPT_P-3_ 6 AA (Gln→Nva) revealed that ΔΔΔG_c_ values are mainly positive and that the allosteric networks overlap (Figs. 2 and 3 and *SI Appendix*, Table S1). A positive coupling free energy means that the effect of the mutation is larger in WT PDZ3 than in the mutant, suggesting that WT PDZ3 is optimized for Thr in position P_-2_ and Gln in position P_-3_, when binding to CRIPT. Furthermore, we found a couple of residues unique for the respective allosteric network; for example, position P_-3_ is coupled to F400 in the α_3_ helix and it also displays a different pattern of ΔΔΔG_c_ values in the β_2_β_3_ loop as compared to position P_-2_. However, most of the reported energetically coupled residues are found within the proximity of the binding pocket, similarly to previous findings (22) (*SI Appendix*, Fig. S6). In general, CRIPT_P-2_ 6 AA reports stronger energetic coupling in comparison with CRIPT_P-3_ 6 AA in PDZ3. This is expected since the mutated hydroxyl group at P_-2_ forms a direct contact to the conserved His372 in PDZ3. Furthermore, the hydroxyl group in CRIPT_P-2_ 6 AA also has stronger energetic coupling to residues in PDZ3 compared with the γ-methyl group of residue P_-2_, mutated in a previous study [Thr→Ser, peptide denoted CRIPT_P-2(Ser)_ 6 AA] (22) (*SI Appendix*, Fig. S6*A* and *C*). This result, while not surprising, clearly shows that the smaller and more specific the perturbation, the more precise is the mapping of coupling free energies and interpretation of allosteric propagation in PDZ3 and other proteins.

### The Allosteric Networks in PSG Are Distinct from Those in Isolated PDZ3.

Binding of peptide ligands to PDZ3 can be analyzed by single exponential kinetics and is therefore well described by a simple two-state binding mechanism (22, 26). However, binding of CRIPT 6 AA to the PSG is double-exponential, suggesting a more complicated binding mechanism involving two distinct conformations of the PSG supramodule as previously reported (30). The two conformations (PSG_A_ and PSG_B_) interconvert on a slow timescale such that binding of CRIPT to both conformations occurs in parallel and is faster than the conformational change between PSG_A_ and PSG_B_ under the conditions in our stopped-flow experiments. Thus, binding to PSG_A_ and PSG_B_, respectively, results in two observed kinetic phases that reflect binding to the respective conformation. Furthermore, we previously demonstrated that the two PSG conformations have similar affinity for CRIPT and showed that the fast kinetic phase, both in association and dissociation experiments, corresponds to one of the conformations (PSG_A_). In the present study, this is the conformation we probe with regard to allosteric networks. In principle we could calculate ΔΔΔG_c_ values for PSG_B_ as well, but rate constants for the slow phase have larger errors (30). Thus, kinetic traces from experiments with CRIPT 6 AA and PSG were fitted to a double-exponential function and *k*_*obs*_ values from the fast kinetic phases were used to estimate *k*_*on*_ and *k*_*off*_ from binding and displacement experiments, respectively (*SI Appendix*, Fig. S5 *C* and *D* and Tables S4 and S5).

Fifteen mutations were chosen in PSG, covering different parts of PDZ3: residues in the binding pocket (G322, F325, I327, and E334), residues common to several proposed allosteric networks in PDZ3 (G330, I338, A347, L353, V362, and A376), residues proposed for one or two networks (G335 and R354) (9), and residues in the α_3_ helix (R399, F400, and E401). Like we observed for PDZ3, the two allosteric networks in PSG, one determined for P_-2_ and the other for P_-3_, overlap. However, comparison of PDZ3 and PSG reveals a completely different pattern of ΔΔΔG_c_ values for PDZ3 in the context of the PSG supramodule as compared with the isolated PDZ3 domain (Figs. 2 *A*–*E* and 3 and *SI Appendix*, Table S1 and Dataset S1). Furthermore, while PDZ3 displays mainly positive coupling free energies PSG displays primarily negative values (Figs. 2 *A*–*E* and 3). Another striking difference is the strong coupling to α_3_ in PSG, which is not observed for PDZ3. Overall, PDZ3 has higher ΔΔΔG_c_ values around the binding pocket, whereas PSG has higher ΔΔΔG_c_ values among residues that are more globally distributed in the PDZ3 domain, for example in the β_1_β_2_ loop, β_2_β_3_ loop, and α_3_ helix. Clearly, the presence of adjacent domains affects the allosteric network in PDZ3, thus suggesting a context-dependent allosteric network in this single protein domain.

### The Allosteric Networks in PDZ3 and PSG Depend on the Length of the CRIPT Peptide.

PDZ3 binds to a type 1 PDZ-binding motif (PBM) defined by a hydrophobic residue at position P_0_ (e.g., Val) and a hydrogen bond donor at P_-2_ (e.g., Thr) (Fig. 1*D*). Residues outside the classical PBM influence the binding too, especially P_-5_ (Tyr) in the case of PDZ3:CRIPT (31, 32). However, beyond P_-5_ the affinity is not significantly affected by the length of CRIPT (33). However, it has not been reported if and how coupling free energies in PDZ and PSG are dependent on peptide ligand length. Therefore, we analyzed the effect of peptide length on the resulting allosteric network, as defined by ΔΔΔG_c_ values, for both PDZ3 and PSG. We report data for CRIPT_P-3_ 15 AA (Fig. 3 and *SI Appendix*, Tables S6 and S7) but could not complete the analysis for CRIPT_P-2_ 15 AA since the kinetic phases were too fast for reliable stopped-flow experiments. Generally, the coupling free energies were weaker for CRIPT 15 AA compared with CRIPT 6 AA (Fig. 3). The pattern was observed in both PDZ3 and PSG. Interestingly, the two networks contain both positive and negative coupling free energies, which could arise from lost selectivity and broader binding profile (34). Therefore, the allosteric networks in PDZ3 and PSG upon binding of CRIPT 15 AA appear more similar (Figs. 2 *C* and *F* and 3). The weaker coupling free energy for CRIPT_P-3_ 15 AA may in part be related to the fact that the dansyl in CRIPT 6 AA sits closer to the PSG and potentially modulates the network by inducing subtle structural changes in the bound state. In fact, CRIPT 6 AA can sense the transition between PSG_A_ and PSG_B_, whereas CRIPT 15 AA cannot (30). Since we observe the pattern in both PDZ3 and PSG we can eliminate the concern that the five extra Trp residues in the SH3–GK tandem will disturb the F337W–dansyl signal pair. However, overall the data suggest that the weaker coupling free energy is a consequence of the 15 AA CRIPT. High coupling free energies are independent of perturbation origin, as CRIPT_P-2_ and CRIPT_P-3_ 6 AA report networks with similar coupling patterns, for example ΔΔΔG_c_ values from G322A and V362A for PSG and I327V, G330A, and A376G for PDZ3. However, high coupling free energies seem to be affected by peptide length. High negative coupling is reported for G322A and V362A in PSG:CRIPT_P-3_ 6 AA but is not present for CRIPT_P-3_ 15 AA (Fig. 3). Thus, the size of the peptide ligand influences the allosteric network, underscoring the extraordinary sensitivity of the energetic connectivity among residues in this protein–ligand complex.

### Monotonic Relationships between the Allosteric Networks.

**Fig. 4.**
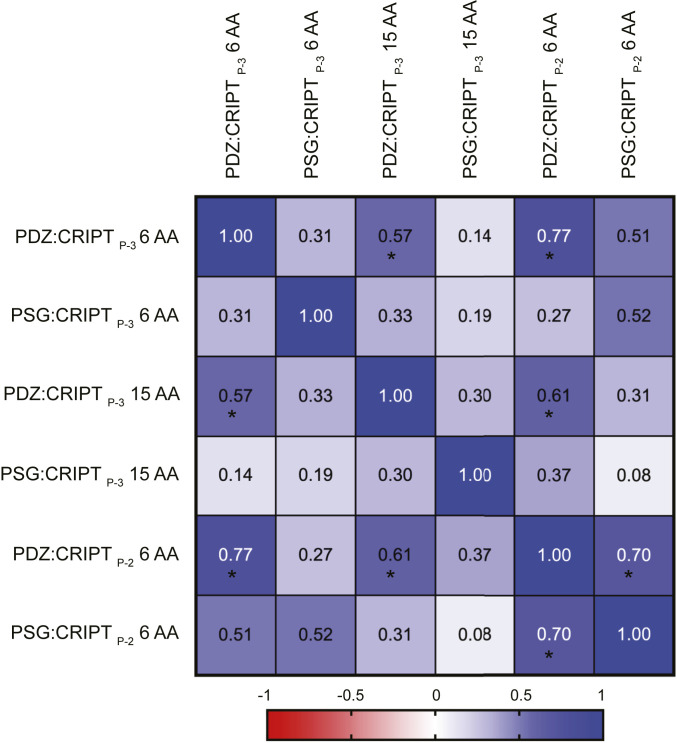
Spearman correlation analysis of the six mapped allosteric networks involving PDZ3. The diagram reports Spearman rank correlation values for six allosteric networks defined by pairwise coupling free energies between residues in PDZ3 and CRIPT. The single domain PDZ3 or PSG was mapped by 14 point mutations in PDZ3 combined with 2 point mutations in CRIPT (Thr_-2_ → Abu or Gln_-3_ → Nva) and for two different CRIPTs, a 6-mer or a 15-mer (CRIPT_P-2_ 6 AA, CRIPT_P-3_ 6 AA or CRIPT_P-3_ 15 AA). The color code going from dark blue (positive) to red (negative) shows the direction and strength of any monotonic relationship. A correlation is marked by * when the *P* value is <0.05.

In an attempt to quantify any correlation among the coupling free energies in the different datasets we calculated Spearman correlation coefficients and plotted them in a heat map (Fig. 4 and *SI Appendix*, Fig. S7). We computed both Spearman and Pearson correlations but choose to report the Spearman correlation as we cannot assume linearity and normal distribution between the variables. With the Spearman analysis and the associated heat map we can visualize the allosteric networks (ranked coupling free energy values) that have a monotonic relationship. Fourteen (Fig. 4) or 22 (*SI Appendix*, Fig. S7) out of 32 mutations in PDZ3 were included in the correlation analysis to allow direct comparison with previously published data (22). Allosteric networks in PDZ3 were probed with four different CRIPT 6 AA peptides (*SI Appendix*, Fig. S7*A*). None of the networks show a perfect correlation to each other (*r* = 1). However, some networks show a monotonic relationship (1 > *r* > 0), with a *P* value lower than 0.05, suggesting that the correlation is not due to random sampling. For example, the allosteric network probed with CRIPT_P-3_ 6 AA shows monotonic relationships to all networks (*P* < 0.05), whereas CRIPT_P-2_ 6 AA only shows monotonic relationship to CRIPT_P-3_ 6 AA and CRIPT_P0_ 6 AA. Note that no relationship is seen between the allosteric networks defined by CRIPT_P-2_ 6 AA and CRIPT_P-2(Ser)_ 6 AA, that is, deriving from the methyl group and the hydroxyl of Thr_-2_, respectively. This can be explained by the contribution from the hydroxyl group, which has a direct interaction to the side chain of His372 (35) and underscores that the character of perturbation, not only residue, is important for the resulting allosteric network. Thus, a general Ala scanning would likely yield a distinct network for position −2 in the peptide. To further visualize correlations between allosteric networks, we quantified monotonic relationships by reanalyzing previous data from five sets of coupling free energy determined for SAP97 PDZ2 and an engineered circular permutated (cp) SAP97 PDZ2. Only one monotonic relationship was found for these datasets, between SAP97 PDZ2:GluR-A_P-2_ 9 AA and cp SAP97 PDZ2:GluR-A_P-2_ 9 AA, both involving the Thr-2 methyl group (25, 36) (*SI Appendix*, Fig. S7*B*). The authors highlighted that stronger coupling free energies were reported for PDZ3 as compared with the structurally similar SAP97 PDZ2 and cp SAP97 PDZ2, thus suggesting that side chains rather than backbone topology govern the magnitude of the allosteric network (25). This is further supported by our data for PDZ3 with CRIPT_P-2_ 6 AA and CRIPT_P-2(Ser)_ 6 AA (*SI Appendix*, Figs. S6 *A* and *C* and S7*A*).

Finally, we applied the Spearman correlation analysis to the six allosteric networks obtained from PDZ3:CRIPT_P-2_ 6 AA, PDZ3:CRIPT_P-3_ 6 AA, PDZ3:CRIPT_P-3_ 15 AA, PSG:CRIPT_P-2_ 6 AA, PSG:CRIPT_P-3_ 6 AA, and PSG:CRIPT_P-3_ 15 AA and including 14 point mutants of PDZ3 (excluding F400A). Four Spearman correlations with *P* < 0.05 were found, where networks involving the γ-methyl of Thr-2 (PDZ3:CRIPT_P-2_ 6 AA) are part of three monotonic relationships (Fig. 4). These three correlations include positions displaying mainly positive or neutral coupling free energies. On the other hand, the PSG:CRIPT_P-3_ 6 AA allosteric network (i.e., emanating from the side chain amide of Gln-3 in CRIPT) has only negative coupling free energies and shows no strong correlation to other allosteric networks, although a trend is present between the PSG:CRIPT_P-3_ 6 AA and PSG:CRIPT_P-2_ 6 AA networks (*P* = 0.06). Taken together, the analysis of correlations between the allosteric networks supports the notion that the magnitude of the coupling free energies in protein domains is governed by side-chain chemistry (sequence) rather than topology, and that they are highly sensitive to supertertiary structure.

### Correlation between Coupling Free Energy and Distance Is Different for PDZ3 and PSG.

**Fig. 5.**
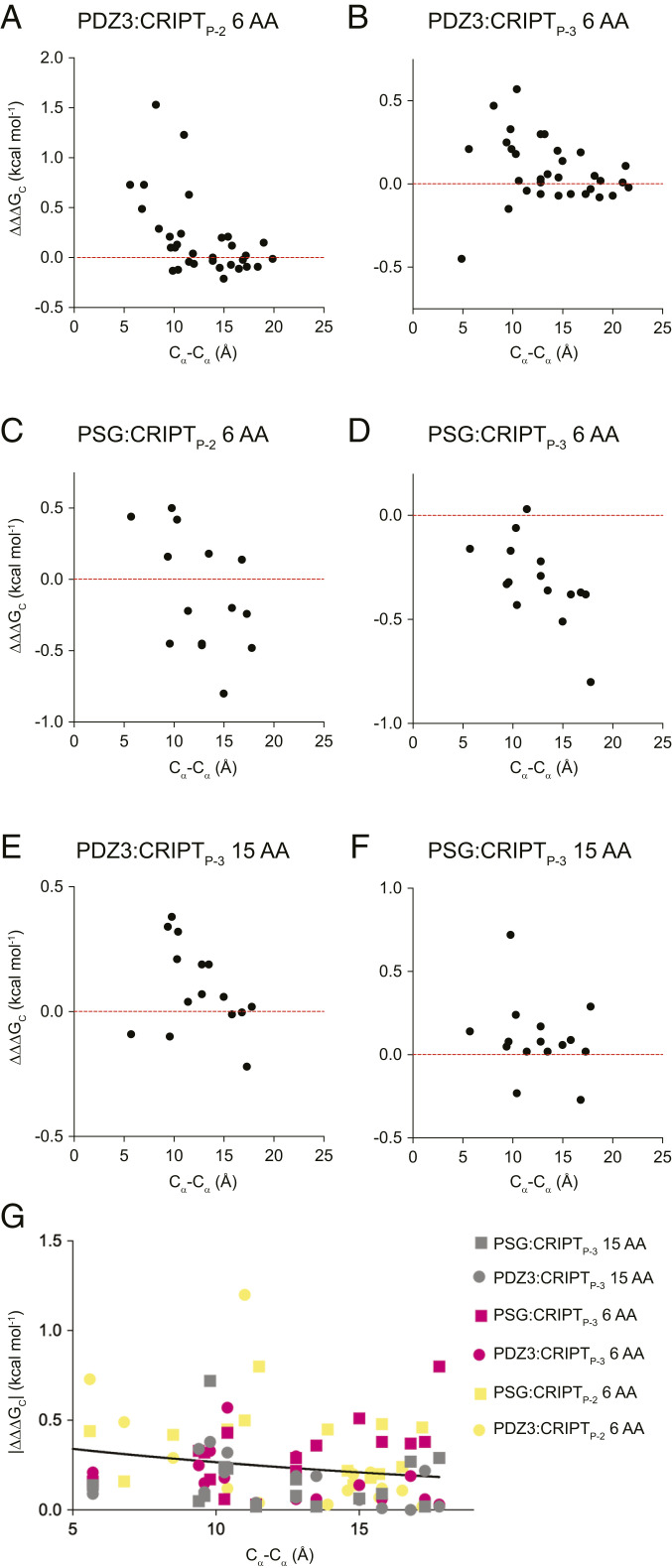
Distance dependence of coupling free energies between CRIPT and PDZ3 or PSG. The coupling free energies were plotted versus C_α_C_α_ distance between the mutated residues in PDZ3 and CRIPT, respectively, for the six different allosteric networks: (*A*) PDZ3:CRIPT_P-2_ 6 AA, (*B*) PDZ3:CRIPT_P-3_ 6 AA, (*C*) PSG:CRIPT_P-2_ 6 AA, (*D*) PSG:CRIPT_P-3_ 6 AA, (*E*) PDZ3:CRIPT_P-3_ 15 AA, and (*F*) PSG:CRIPT_P-3_ 15 AA. The red horizontal line divides negative from positive coupling free energies. (*G*) The absolute number of all coupling free energies |∆∆∆G_c_| from our double-mutant cycles in PDZ3 and PSG show no significant distance dependence, as illustrated by the exponential fit (distance constant d_c_ = 21 ± 11 Å).

Another striking difference between our datasets is the distance dependence of ΔΔΔG_c_ values. In the trivial case with a nonallosteric globular protein we would expect coupling free energies to decrease with distance between the two mutations. For example, two directly interacting residues (distance <5 Å) will couple whereas residues separated by 20 Å will not. The coupling free energy between residue pairs in the PDZ3 and PSG complexes was plotted against C_α_–C_α_ distance (Fig. 5). For PDZ3:CRIPT a rather clear trend was observed in which residues with high coupling free energy were located closer to the binding pocket, both for the CRIPT 6 AA and CRIPT 15 AA (Fig. 5*A*, *B*, and *E*). On the other hand, for PSG:CRIPT 6 AA we observed a peculiar inverse trend where proximal residues displayed ΔΔΔG_c_ values close to zero or slightly positive whereas distal residues displayed larger and negative ΔΔΔG_c_ values (Fig. 5 *C* and *D*). For PSG:CRIPT 15 AA the magnitude of ΔΔΔG_c_ values appeared independent of distance. In a previous study with PDZ3:CRIPT 6 AA and two other peptide mutations (P_-2_ Thr→Ser and P_0_ Val→Abu) no clear trend was observed between coupling free energy and distance (22). Altogether, the distance dependence of coupling free energies in PDZ3:CRIPT complexes are context-dependent, both with regard to adjacent domains, length of peptide ligand, and the precise mutation in the peptide. Overall, this variation supports our earlier notion that sequence rather than topology determines allosteric networks as quantified by coupling free energies (22, 25, 37). The noticeable change in coupling free energies from positive in PDZ3 to mainly negative in PSG underscores the hypothesis that residues rather than backbone transfer the coupling free energy (25). PSG:CRIPT_P-2_ 6 AA reports positive coupling free energies for residues located next to the P_-2_ position whereas the rest of the residues have negative ΔΔΔG_c_ values. Strikingly, the PSG:CRIPT_P-3_ 6 AA interaction only reports negative and neutral ΔΔΔG_c_ values. Negatively coupled residues are primarily located at the interface between the PDZ3 and SH3 domains in the supramodule PSG (38). Naganathan and coworkers showed, in a reanalysis of 49 structural perturbation datasets in 28 different proteins, a universal pattern in which the effect of a perturbation decays exponentially with distance from the perturbation (39). Therefore, we subjected our dataset to the same analysis and plotted the numerical (absolute) value of ΔΔΔG_c_ against distance for all six datasets and fitted to an exponential function (Fig. 5*G*). No clear trend was observed between distance and magnitude of perturbation (|ΔΔΔG_c_|) from our double-mutant cycle analysis, consistent with a sequence-dependent allosteric behavior.

### Experimental error of ΔΔΔG_c_ values.

Our conclusions depend on accurate and rather precise ΔΔΔG_c_ values. The errors reported for ΔΔΔG_c_ are propagated fitting errors from the kinetic experiments, except for eight interactions with WT PDZ or PSG, which are reported as SDs from replicate experiments (*n* = 2 to 6) (Dataset S1 *A*–*D*). In order to further validate the accuracy of the data we repeated the double-mutant cycle two times for five different residue pairs displaying a range of coupling free energies spanning from negative to positive (*SI Appendix*, Fig. S8). ΔΔΔG_c_ values for these five double-mutant cycles varied within 0.11 and 0.24 kcal/mol, with SDs (0.057 to 0.13 kcal/mol, *n* = 3) in the same order as our propagated errors, demonstrating that our conclusions are robust to the error in ΔΔΔG_c_. In accordance with these errors the color code of ΔΔΔG_c_ values in the graphics is divided into 0.20-kcal/mol intervals. Note that the error in ΔΔΔG_c_ is absolute and not relative.

## Discussion

While it is well established that many biological processes are finely regulated by the allosteric nature of protein domains, a quantitative description of the energetic communication between two distinct sites in a protein is still very challenging. Nevertheless, unveiling the subset of residues that form a physically connected network between two distinct sites can have multiple applications, for example for protein engineering or finding a more effective and selective drug discovery approach, as allosteric sites are less conserved than the primary binding pocket within a given protein family (40). In the case of PDZ domains, it has been shown how they represent interesting drug targets in a range of diseases including stroke and cancer. However, there is no approved drug for a PDZ domain due to their promiscuity, which is manifested as binding to several similar ligands through C-terminal recognition. The presence of an evolutionarily conserved sparse allosteric network in PDZ3 was proposed two decades ago based on multiple sequence alignments and statistical coupling analysis, showing an interresidue communication from the binding pocket to a proposed allosteric site located around β_1_ and β_2_ (8). However, there is still limited application of the allosteric network in PDZ engineering and drug design as the presence of one conserved allosteric network in the PDZ family was challenged by various in silico and experimental approaches (*SI Appendix*, Fig. S1). In fact, the growing number of distinct allosteric networks in PDZ3 suggested that the choice of method influences the identity of the network (9) (*SI Appendix*, Fig. S1).

### PDZ Domains Studied in Isolation.

PDZ domains play a role in protein targeting and protein complex assembly and are often found in multidomain proteins. Despite this, most in vitro studies use single PDZ domains to reduce complexity. Moreover, inspection of the subset of allosteric networks for PDZ3 reported in *SI Appendix*, Fig. S1 shows that eight different studies have used different construct length of the PDZ3 domain, which may also affect the mapped allosteric network. One example is the α_3_ helix, which was only included in six constructs, although it is important for binding affinity (15), folding (28), and dynamics (29). PDZ3 shares overall fold with other PDZ domains, but the α_3_ helix is exclusive for PDZ3 domains from MAGUK proteins. Thus, methods that rely on a multiple sequence alignment of all PDZ domains cannot include the α_3_ helix, which might be a limitation.

### Supertertiary Structure Affects the Allosteric Network in PDZ3.

In the present study, we addressed the role of supertertiary structure by mapping the allosteric network in isolated PDZ3 as well as in the PSG supramodule. Surprisingly, we observe that not only α_3_ affects the allosteric network in PDZ3, but there is a substantial difference when the domain is studied in isolation as compared to when it is part of the PSG supramodule. This finding is in line with previous studies indicating that the allostery is mediated through interdomain communication by protein–protein interactions (15, 18).

**Fig. 6.**
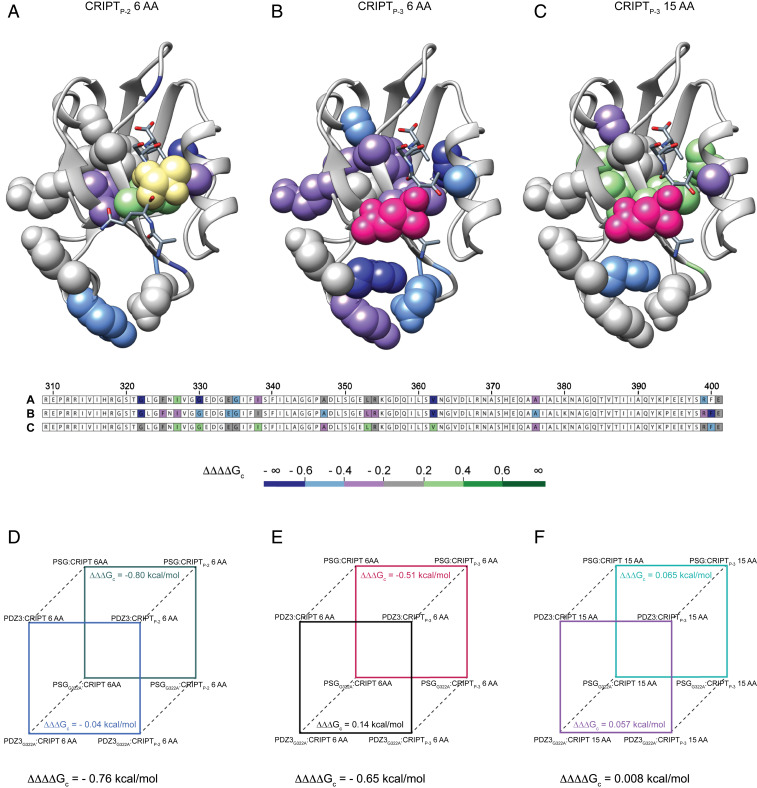
Differences between the allosteric networks in PDZ3 and PSG. The coupling free energies obtained with the single PDZ3 domain were subtracted from those of PSG for the corresponding positions to visualize the effect of SH3–GK on the allosteric network in PDZ3 upon CRIPT binding. The three panels show the resulting ∆∆∆∆G_c_ values (= ∆∆∆G_c_^PSG^ − ∆∆∆G_c_^PDZ3^) for the respective CRIPT residue (Thr_-2_ or Gln_-3_) and peptide and for each tested residue in PDZ3, mapped onto the PDZ3:CRIPT complex. (*A*) CRIPT_P-2_ 6 AA, (*B*) CRIPT_P-3_ 6 AA, and (*C*) CRIPT_P-3_ 15 AA. The residue probed by mutation in CRIPT is shown as yellow (P_-2_, Thr_-2_ → Abu) or pink (P_-3_, Gln_-3_ → Nva) spheres. The side chains probed by mutation in PDZ3 and PSG are depicted as spheres with a color code corresponding to the difference in coupling free energy (∆∆∆∆G_c_) between PSG and PDZ3 for the respective side chain. Residues with gray color show a similar response in PDZ3 and PSG to the mutational perturbation of the allosteric network. (*D*–*F*) To illustrate how the ∆∆∆∆G_c_ values were calculated, thermodynamic cubes are shown for three cases representing *A*–*C*. Thus, each ∆∆∆∆G_c_ value is based on three perturbations: (*D*) Gly322→Ala, Thr_-2_→Abu (in 6-mer CRIPT) and PDZ3→PSG, (*E*) Gly322→Ala, Gln_-3_→Nva (in 6-mer CRIPT) and PDZ3→PSG, and (*F*) Gly322→Ala, Gln_-3_→Nva (in 15-mer CRIPT) and PDZ3→PSG.

To visualize how the presence of the SH3 and GK domains affects the allosteric network in PDZ3 (Fig. 1*C*), we calculated ∆∆∆∆G_c_ values by subtracting ∆∆∆G_c_ for PDZ3 from ∆∆∆G_c_ for PSG (Fig. 6 and *SI Appendix*, Table S8 and Dataset S1*E*). ∆∆∆∆G_c_ values were mapped onto the structure of PDZ3 for CRIPT_P-2_ 6 AA, CRIPT_P-3_ 6 AA, and CRIPT_P-3_ 15 AA. The allosteric networks appear different, especially at the α_3_ helix and β_1_β_2_ and β_2_β_3_ loops, as represented by the dark blue color (Fig. 6 *A* and *B*). In accordance with previous findings (18, 38) we propose that the allosteric network propagates to new sites in the context of the supramodule. Zhang et al., showed that the PSG from PSD-95 changes from compact to extended conformation upon CRIPT binding, which disturbs PDZ3:SH3 Interdomain interactions at the β_1_β_2_ loop, β_2_ sheet, and α_2_ and α_3_ helix in PDZ3 (38). The interdomain dynamics of PDZ3:SH3 indicates that the PSG supramodule is flexible and changes interdomain conformation in response to ligand binding (38), which is supported by our results and other recent findings showing that intramolecular domain dynamics regulate the binding properties and conformations of the PSG supramodule (18). However, the interactions mediated by the β_1_β_2_ and β_2_β_3_ loops could be a general feature for PDZ supertertiary organization as reported for the PDZ1-2 tandem (41). Taken together, the data show the importance of three structural elements for inter- and intradomain structural conformation and communication: the α_3_ helix and the β_1_β_2_ and β_2_β_3_ loops.

### The Significance of Positive ΔΔΔG_c_ Values for Allostery and Selectivity.

Selectivity of single domains can be affected by long-range allosteric networks as shown for SH3 (42, 43) as well as PDZ; for example, PDZ1 from PTP-BL modulates the binding of ligands to the adjacent PDZ2 (42). We have previously argued that positive ΔΔΔG_c_ values are a signature for optimization of binding a certain ligand (22). Such a trend was reported here for the allosteric networks with CRIPT_P-2_ 6 AA, CRIPT_P-3_ 6 AA, and CRIPT_P-3_ 15 AA in PDZ3. Thus, a positive sign of ΔΔΔG_c_ means that a mutation in PDZ3 will result in a smaller effect of the second mutation (in CRIPT) (Fig. 1*B*). In other words, mutation disrupts an optimized interaction network and relaxes the system with regard to further perturbation. In contrast to the mainly positive ΔΔΔG_c_ values for PDZ3, surprisingly, we observe primarily negative ΔΔΔG_c_ values for the allosteric network in PSG probed by CRIPT_P-3_ 6 AA, suggesting a nonoptimized system. A tendency for negative ΔΔΔG_c_ values was recently reported for an SH3 domain (34). Thus, the presence of allosteric networks may not necessarily be linked with binding selectivity but may also trace the communication pathways between energetically interacting domains in supramodules. Analogously, the negative values in PDZ3 indicate a complex role for such domains with multiple binding partners in the presence of the α_3_ extension or the SH3–GK domains (15, 44). We note in this context that a more complex supertertiary structure may have several native or nonnative states, which would affect the allosteric coupling (45).

### Mechanistic Aspects of the Allosteric Networks.

Classically, allostery was associated with relatively large conformational changes in the protein structure (1, 2). Later work implicated dynamics in allosteric mechanisms (3, 7, 11) and thereby a prominent role for entropy. Wand and coworkers demonstrated a correlation between fast side-chain dynamics and conformational entropy in protein–ligand interactions (46) and showed that contributions from side-chain dynamics in some cases make a considerable contribution to ΔG (47). Lee and coworkers have studied both the PSG and PDZ3 using NMR methods and proposed that interdomain allostery in PSG is mediated by structural changes in flexible regions (38), whereas changes in conformational entropy involving side-chain dynamics underlie intradomain allostery within the PDZ3 domain. This model was challenged by Kumawat and Chakrabarty, who argued, based on molecular dynamics simulations, that a redistribution of electrostatic interactions in PDZ3 upon ligand binding can explain the observed energetic networks (17). Both models are consistent with the lack of observable conformational changes in PDZ3 upon binding of peptide ligand (35) and it is plausible that conformational entropy and redistribution of interactions conspire to reshape the binding energy landscape upon perturbation.

Our double-mutant cycle approach involves equilibrium free energies. Consequently, the resulting ΔΔΔG_c_ values report whether or not certain side chains are energetically connected. However, this type of analysis, while being highly sensitive, cannot assess if the energetic contribution is entropic, enthalpic, or both. While our data cannot distinguish mechanisms, they show that a necessary condition is that it is sensitive to perturbation. To put this in context, protein folding has proven robust to mutation to the extent that homologous proteins with similar topology (including PDZ domains) usually follow the same folding mechanism (48). On the other hand, the allosteric network in PDZ3 is easily rewired by perturbation. Conformational entropy (29) as well as redistributions of distinct populations of energetically similar electrostatic networks (17) may both be sensitive to perturbations and thus consistent with the results in the present paper.

### Concluding Remarks: What Is next for Allosteric Networks in PDZ3?

Is there one unique or multiple allosteric networks in PDZ3? Allosteric networks have been mapped in PDZ3 using different primary structures as input and analyzed by various methods (*SI Appendix*, Fig. S1), which has hampered a direct comparison. We demonstrate unequivocally that the allosteric network as defined by ΔΔΔG_c_ values is different for PDZ3 present as a single domain and in a supertertiary structure, the PSG supramodule. Therefore, we encourage using multidomain constructs in experimental and theoretical studies on intradomain allosteric networks, in particular when the domain is present in a supertertiary structure, like PDZ3. Transiting from single to multiple domains involves experimental challenges and previous focus on single domains may have hampered drug discovery. Nevertheless, the complexity and dynamics of multiple domains can offer new opportunities to control protein–protein interactions (49) and hopefully increase the possibilities to profit from allosteric networks in PDZ drug design.

## Methods

### Protein Expression and Purification.

WT PSD-95 PDZ3-SH3-GK (PSG), PDZ3 domain and mutants (all pseudo WTs with an engineered F337W in the PDZ3 domain) were encoded in modified pRSET vectors (Invitrogen) transformed into *Escherichia coli* BL21(DE3) pLys cells (Invitrogen). Cells were grown in lysogeny broth medium at 37 °C and overexpression of protein was induced with 1 mM isopropyl β-d-1-thiogalactopyranoside overnight at 18 °C. Cells were harvested at 4 °C and pellet resolubilized in 50 mM Tris, pH 7.8, and 10% glycerol (and including 100 mM NaCl for PSG) and stored at −20 °C. Pellets were sonicated twice for 4 min followed by centrifugation at 50,000 × *g* at 4 °C for 1 h. Supernatants were filtered and added to a preequilibrated (50 mM Tris and 10% glycerol [including 100 mM NaCl and 0.5 mM DTT for PSG]) Nickel Sepharose Fast Flow column (GE Healthcare). Proteins were eluted with 250 mM imidazole and dialyzed overnight into 50 mM Tris pH 7.8 (PDZ3) or 50 mM Tris, 2 mM DTT, 100 mM NaCl, and 10% glycerol (PSG). PDZ3 or mutants were loaded onto a QS Sepharose column for further purification and eluted with a 500 mM NaCl gradient. PSG or mutants were concentrated and further purified using size-exclusion chromatography (S-100; GE Healthcare). Protein purity was quantified by sodium dodecyl sulfate polyacrylamide gel electrophoresis and identity checked by matrix-assisted laser desorption ionization time-of-flight mass spectrometry. Concentration of proteins were estimated from the absorbance at 280 nm. CD scans of PDZ3, PSG, and mutants were performed to determine if all proteins were folded. All CD experiments were performed in 50 mM sodium phosphate, pH 7.45, and 21 mM KCl (I = 150) at 10 °C on a JASCO 1500 spectropolarimeter using the average of five scans. Far-ultraviolet spectra were recorded from 260 nm to 200 nm with 10 μM protein.

### Peptide Synthesis.

Dansyl-labeled CRIPT peptides were ordered (Zhejiang Ontores Biotechnologies) or synthesized manually by solid-phase peptide synthesis as described previously (33). Concentrations were measured at 340 nm using an extinction coefficient of 3,400 M^−1^⋅cm^−1^ (50). Based on carbon-chain length but without functional groups (OH and amide) unnatural amino acids were introduced at position P_-2_: Thr→Abu (aminobutyric acid, R = CH_2_CH_3_) and position P_-3_: Gln→Nva (norvaline, R = CH_2_CH_2_CH_3_).

### Kinetic Experiments.

All kinetic experiments were performed in 50 mM sodium phosphate, pH 7.45, and 21 mM KCl (I = 150) at 10 °C (including 0.5 mM Tris-2-carboxyethyl-phosphine [TCEP] for experiments with PSG). Binding and dissociation experiments were performed using an upgraded SX-17 MV stopped-flow spectrophotometer (Applied Photophysics) and carried out as described previously (51).

Association rate constants (*k*_on_) were obtained from binding experiments under conditions approaching pseudo first order at high CRIPT concentrations (dansylated) ([PDZ or PSG] < [CRIPT]). Thus, PDZ (1 μM) was mixed rapidly with increasing concentrations of dansylated CRIPT ligand (2 to 20 μM of CRIPT 6 AA, CRIPT_P-2_ 6 AA, CRIPT_P-3_ 6 AA, CRIPT 15 AA, CRIPT_P-2_ 15 AA, or CRIPT_P-3_ 6 AA). Excitation was at 280 nm and emission measured around 330 nm (using a 330 ± 25 nm interference filter). Kinetic traces (minimum of five) were recorded, averaged, and fitted to a single exponential function (a double exponential was used for PSG with CRIPT 6 AA, CRIPT_P-2_ 6 AA, and CRIPT_P-3_ 6 AA) consistent with a two-state bimolecular association/dissociation mechanism. The fitting yielded observed rate constants (*k*_*obs*_) for each CRIPT concentration. A plot with *k*_*obs*_ versus CRIPT concentration was obtained and fitted to an equation derived for a second-order bimolecular association reaction to account for the deviation from linearity at low CRIPT concentrations:

$$k_{obs}={\left(k_{on}^{2}\cdot {\left(\left[CRIPT\right]-{\left[PDZ3\right]}_{0}\right)}^{2}+k_{off}^{2}+2\cdot k_{on}k_{off}\left(\left[CRIPT\right]+{\left[PDZ3\right]}_{0}\right)\right)}^{0.5},$$

where [PDZ3]_0_ and [CRIPT] are the initial protein and peptide concentrations. While the association rate constant (*k*_*on*_) was obtained from fitting of this equation, the dissociation rate constant (*k*_*off*_) was obtained from displacement experiments. PDZ or PSG (2 μM) was mixed with dansylated CRIPT (10 μM) followed by a long incubation (>15 min) to ensure that equilibrium was established. Dansylated CRIPT was displaced from PDZ by mixing with an excess of unlabeled CRIPT (100, 150, or 200 μM). *k*_*off*_ values were estimated from the average of three *k*_*obs*_ determinations at high concentrations of unlabeled CRIPT peptide, where the *k*_*obs*_ values were constant with unlabeled CRIPT concentration. All binding and displacement experiments performed with PSG and CRIPT 6 AA were fitted to double exponential function due to more complex binding mechanism, and *k*_*obs*_ values from the fast phase in the respective experiment were used for further analysis (30).

### ITC.

All ITC experiments were performed in 50 mM sodium phosphate, pH 7.45, and 21 mM KCl (I = 150 mM) at 25 °C, and including 0.5 mM TCEP for experiments with PSG, in an iTC200 instrument (Malvern). CRIPT and PDZ or PSG were dialyzed overnight in the same buffer to minimize artifacts from buffer mismatch in the experiment. CRIPT was titrated (16 to 19 injections) into a cell with PDZ or PSG, and saturation was obtained by having 10 to 20 times higher concentration of CRIPT in the syringe, resulting in a roughly twofold excess of CRIPT at the end of the titration. Baseline corrections were performed to minimize the chi value in curve fitting. Fitting of data were performed using the iTC200 software.

### Calculation of coupling free energies.

Coupling free energies were calculated from *K*_d_ values determined by the ratio *k*_*off*_/*k*_*on*_ for all four complexes in the double mutant cycle (Fig. 1*B* and Dataset S1 *A*–*D*) as described (21, 22). Concisely, four complexes are characterized for each double-mutant cycle: 1 (WT), 2 (single mutation in PDZ or PSG), 3 (single mutation in CRIPT), and 4 (single mutation in PDZ/PSG and CRIPT). The change in free energy upon single point mutation in PDZ, can be measured by calculating the difference in energy from complex 1–2 (Eq. **1**). Similarly, the energy change upon single point mutation in PDZ bound to mutated CRIPT, can be measured by calculating the difference in energy from complex 3–4 (Eq. **2**). Therefore, if the residue subjected to single point mutation in PDZ does not interact energetically with the residue mutated in CRIPT, the energy difference upon single point mutation in PDZ will be similar for the two complexes ${\Delta \Delta \text{G}}_{PDZ3_{WT}:CRIPT_{Q}\left(1\right)\to \;PDZ3_{X}:CRIPT_{Q}\left(2\right)}=\;{\Delta \Delta \text{G}}_{PDZ3_{WT}:CRIPT_{Nva}\left(3\right)\;\to \;PDZ3_{X}:CRIPT_{Nva}\left(4\right)}$ and meaning that the coupling free energy $\left(\Delta \Delta \Delta G_{c}\right)$ is zero (Eq. **3**). However, if the residues subjected to single point mutations in PDZ (X) and CRIPT (Nva) interact energetically the calculated $\Delta \Delta \Delta G_{c}\neq 0.$This coupling free energy is then a quantitative measure of the strength of the interaction between the two residues, one in PDZ and one in CRIPT:

$${\Delta \Delta \text{G}}_{PDZ3_{WT}:CRIPT_{Q}\left(1\right)\to \;PDZ3_{X}:CRIPT_{Q}\left(2\right)}=\;RT\cdot \text{ln}\left(\frac{k_{on\left(1\right)}}{k_{off\left(1\right)}}\cdot \frac{k_{off\left(2\right)}}{k_{on\left(2\right)}}\right)$$ [1]

$${\Delta \Delta \text{G}}_{PDZ3_{WT}:CRIPT_{Nva}\left(3\right)\;\to \;PDZ3_{X}:CRIPT_{Nva}\left(4\right)}=\;RT\cdot \text{ln}\left(\frac{k_{on\left(3\right)}}{k_{off\left(3\right)}}\cdot \frac{k_{off\left(4\right)}}{k_{on\left(4\right)}}\right)$$ [2]

$$\Delta \Delta \Delta G_{c}={\Delta \Delta \text{G}}_{PDZ3_{WT}:CRIPT_{Q}\left(1\right)\;\to \;PDZ3_{X}:CRIPT_{Q}\left(2\right)}-\;{\Delta \Delta \text{G}}_{PDZ3_{WT}:CRIPT_{Nva}\left(3\right)\;\to \;PDZ3_{X}:CRIPT_{Nva}\left(4\right)}.$$ [3]

## Supplementary Material

Supplementary File

Supplementary File

## Data Availability

All kinetic rate constants and calculations are provided in Dataset S1. All study data are included in the paper and *SI Appendix*.
