# Supplementary material for: Supertertiary protein structure affects an allosteric network
Source: Proc Natl Acad Sci U S A. 2020 Sep 14;117(39):24294–304. doi: 10.1073/pnas.2007201117 (PMC7533695; doi:10.1073/pnas.2007201117)
Supplement: Supplementary File [file pnas.2007201117.sapp.pdf]

Supplementary material

Supertertiary protein structure affects an allosteric network

Louise Laursen[1], Johanna Kliche[1,2], Stefano Gianni[3]* and Per Jemth[1]*

[1]Department of Medical Biochemistry and Microbiology, Uppsala University, BMC Box 582, SE-75123 Uppsala, Sweden.

[2]Present:address: Department of Chemistry-BMC, Uppsala University, Box 576, SE-751 23 Uppsala, Sweden.

[3]Istituto Pasteur-Fondazione Cenci Bolognetti and Istituto di Biologia e Patologia Molecolari del CNR, Dipartimento di Scienze Biochimiche "A. Rossi Fanelli," Sapienza Università di Roma, 00185 Rome, Italy.

*Corresponding authors: Stefano.Gianni@uniroma1.it; Per.Jemth@imbim.uu.se

www.pnas.org/cgi/doi/10.1073/pnas.2007201117

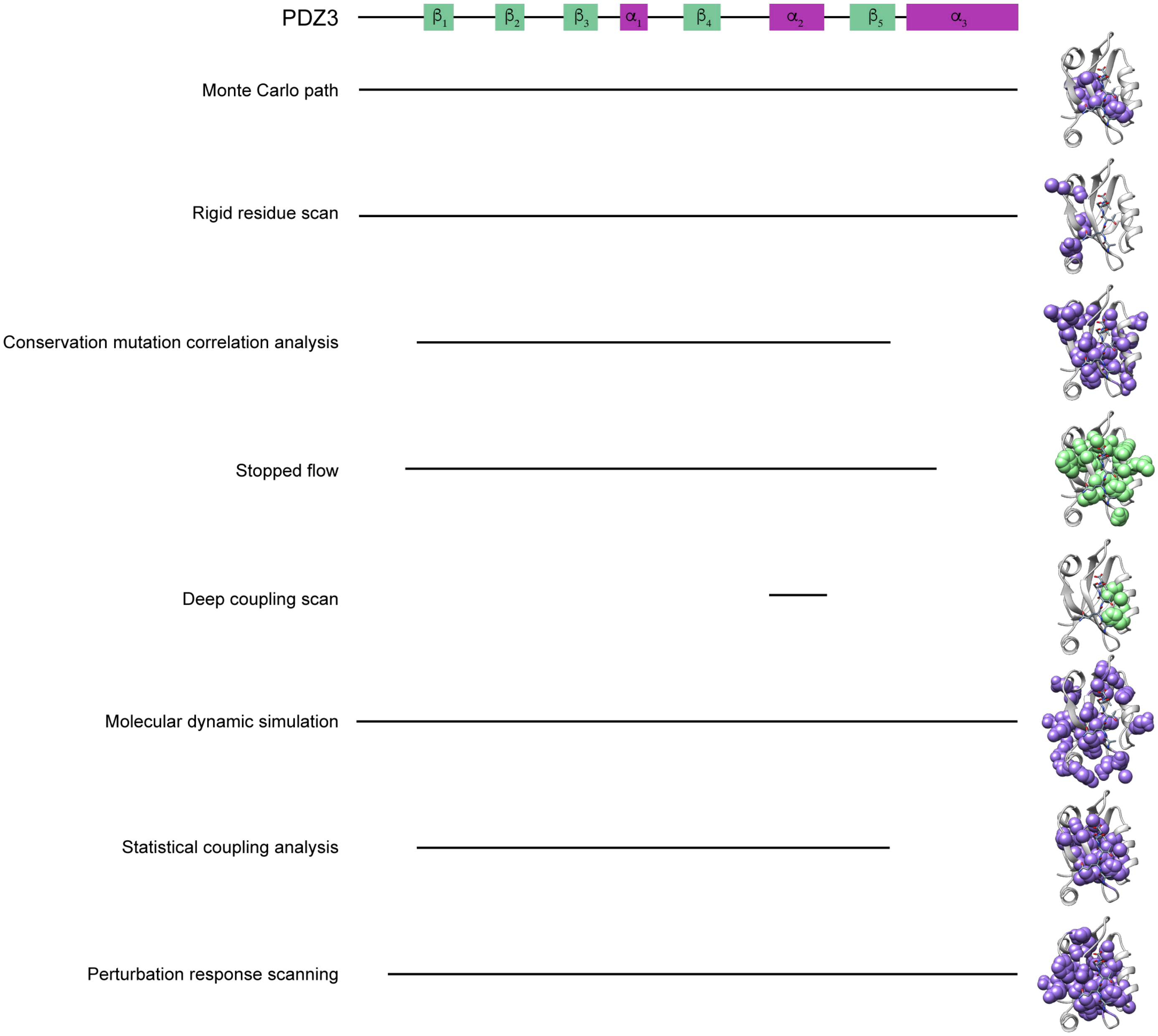

Fig. S1.
Allosteric networks in PDZ3 determined by various methods
Overview showing 8 different allosteric networks reported in PDZ3 using in silico approaches (six examples, purple) and two experimental approaches (green). The illustration highlights the different lengths of PDZ3 constructs used for the different approaches: Monte Carlo path [1], rigid residue scan [2], conservation mutation correlation analysis [3], thermodynamic double mutant cycle by stopped flow [4], deep coupling scan [5], molecular dynamics scan [6], statistical coupling analysis [7], perturbation response scanning [8]. The network illustrations were adapted from Gautier et al [9].

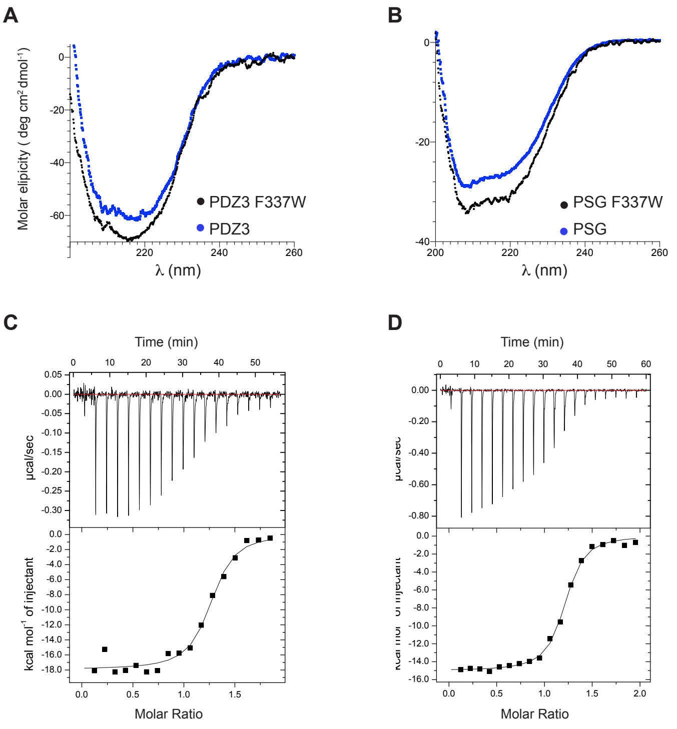

Fig. S2.

F337W in PDZ3 and PSG does not affect secondary structure and affinity of CRIPT

Circular dichroism spectra at 200-260 nm for PDZ3 **A** and PSG **B** were recorded in 50 mM sodium phosphate pH 7.4, 10°C. ITC experiments for CRIPT 6 AA binding to **C** PSG (K_d = 0.14 µM) and **D** PSG with F337W probe (K_d = 0.18 µM) were performed in 50 mM sodium phosphate pH 7.4, 25°C.

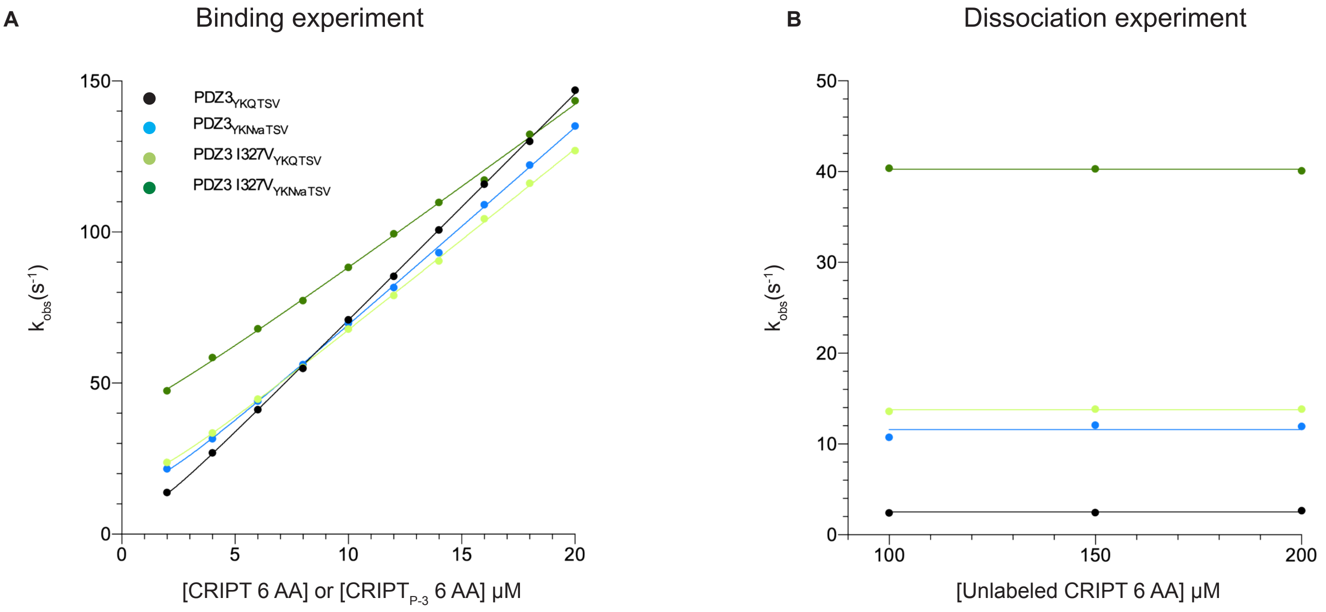

C

●	PDZ3:YKQTSV	k_{on} = 7.0 μM^{-1}s^{-1}	k_{off} = 2.6 s^{-1}	K_d = 0.37 μM
●	PDZ3$_{I327V}$:YKQTSV	k_{on} = 6.1 μM^{-1}s^{-1}	k_{off} = 14 s^{-1}	K_d = 2.3 μM
●	PDZ3:YKNvaTSV	k_{on} = 6.3 μM^{-1}s^{-1}	k_{off} = 11 s^{-1}	K_d = 1.7 μM
●	PDZ3$_{I327V}$:YKNvaTSV	k_{on} = 5.6 μM^{-1}s^{-1}	k_{off} = 40 s^{-1}	K_d = 7.2 μM

D

$$\Delta\Delta\Delta G_c = RT \ln \frac{K_d^{PDZ3:YKNvaTSV} \bullet K_d^{PDZ3(I327V):YKQTSV}}{K_d^{PDZ3:YKQTSV} \bullet K_d^{PDZ3(I327V):YKNvaTSV}}$$

$$= 0.562\ \text{kcal}\bullet\text{mol}^{-1} \frac{1.7\ \mu M \bullet 2.3\ \mu M}{0.37\ \mu M \bullet 7.2\ \mu M} = 0.21\ \text{kcal}\bullet\text{mol}^{-1}$$

Fig. S3.

Calculation example of coupling free energy

Calculation example of coupling free energy ($\Delta\Delta\Delta G_c$) from observed rate constants for WT and I327V PDZ3. **A** Plot showing the linear relationship between observed rate constants and CRIPT 6 AA concentration under the experimental conditions. The association rate constant (k_{on}) is equal to the slope of the curve. **B** The dissociation rate constant (k_{off}) was estimated from a separate displacement experiment. The plot shows observed rate constants from displacement experiments at three concentrations of unlabeled CRIPT 6 AA. Displacement experiments were performed by preincubating PDZ3:CRIPT 6 AA (dansylated) and mixing with 100, 150, or 200 μM unlabeled CRIPT 6 AA. At high concentration the dissociation of labeled CRIPT 6 AA is practically irreversible as shown by the slopes, which are ≈ 0. The average of three observed rate constants was used as an estimate of the dissociation rate constant. **C** The K_d value was calculated from the ratio of the dissociation and association rate constants for the four complexes. **D** Calculation example of coupling free energy ($\Delta\Delta\Delta G_c$) for the double mutant cycle probing the interaction between Gln_{-3} in CRIPT 6 AA and Ile327 in PDZ3. The numbers are usually rounded to two significant figures in tables and figures but in the calculations we use at least three obtained from raw k_{obs} data. Further examples of raw data are shown in Fig. S5.

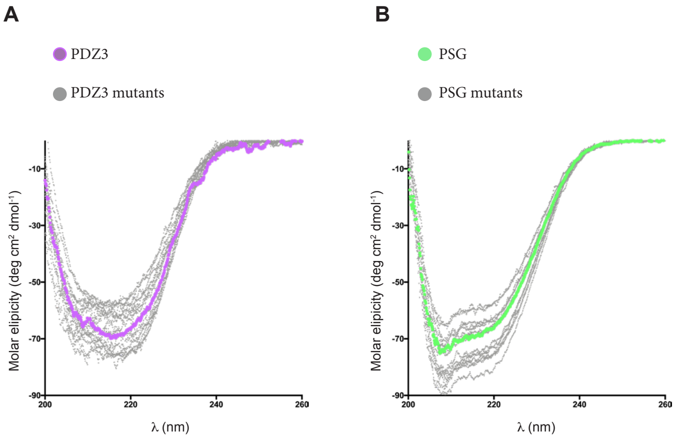

Fig. S4.

The Mutations did not perturb secondary structure of PDZ3 and PSG

CD spectra shown for 15 mutants of PDZ3 **A** and PSG **B**. Color code: PDZ3 WT purple, PSG WT green and mutants grey. The shape of the spectra are similar, suggesting a similar fold. The difference in magnitude likely reflects differences in concentration. Note that errors in the concentration of protein have virtually no effect on the observed rate constants and calculated K_d values. The spectra were recorded in 50 mM Sodium Phosphate pH 7.4, 10°C including 0.5 mM TCEP for PSG.

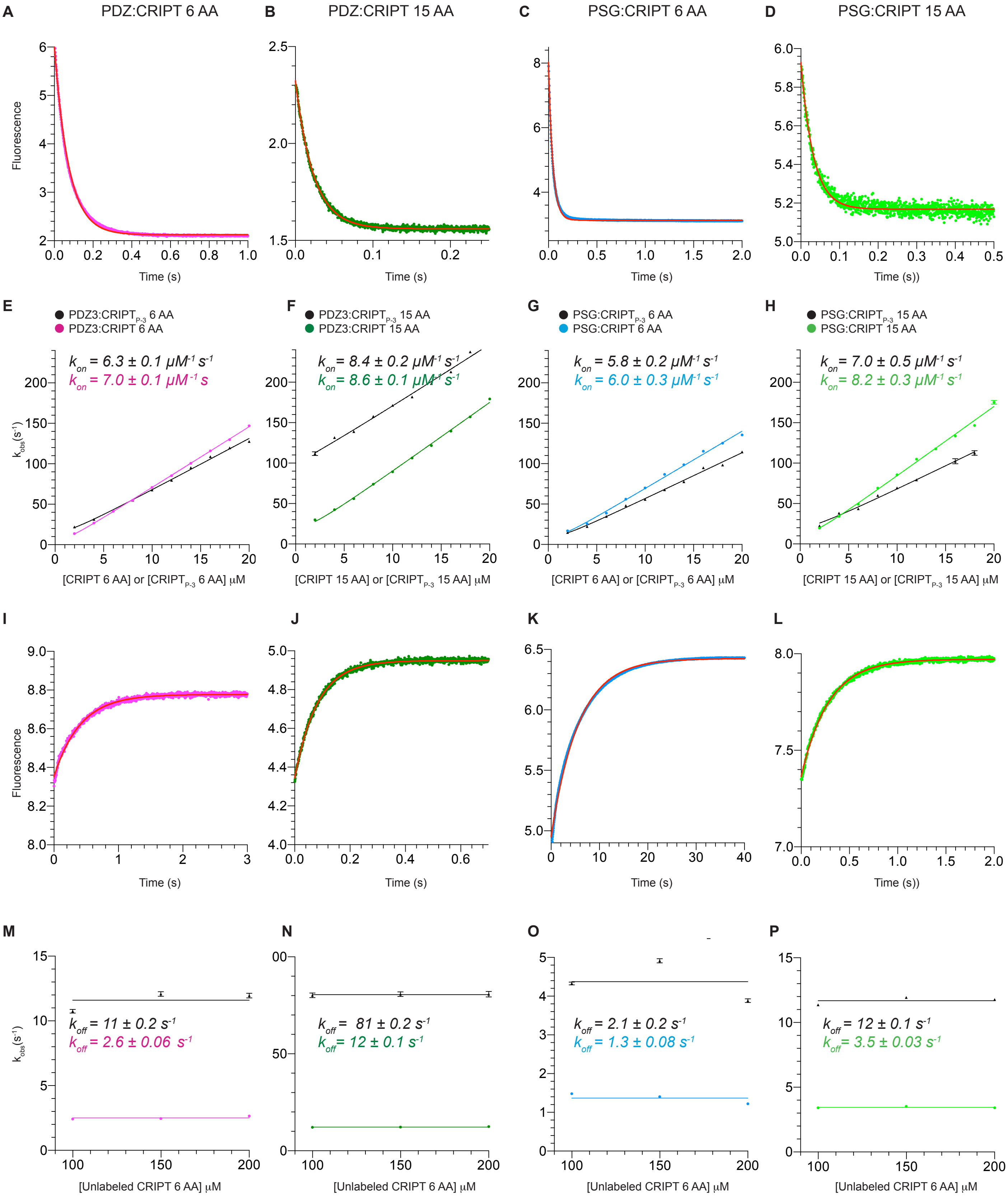
A
PDZ:CRIPT 6 AA
B
PDZ:CRIPT 15 AA
C
PSG:CRIPT 6 AA
D
PSG:CRIPT 15 AA
Fluorescence
Time (s)
Time (s))
E
PDZ3:CRIPT$_{P-3}$ 6 AA
PDZ3:CRIPT 6 AA
k_{on} = 6.3 ± 0.1 μM^{-1} s^{-1}
k_{on} = 7.0 ± 0.1 μM^{-1} s
F
PDZ3:CRIPT$_{P-3}$ 15 AA
PDZ3:CRIPT 15 AA
k_{on} = 8.4 ± 0.2 μM^{-1} s^{-1}
k_{on} = 8.6 ± 0.1 μM^{-1} s^{-1}
G
PSG:CRIPT$_{P-3}$ 6 AA
PSG:CRIPT 6 AA
k_{on} = 5.8 ± 0.2 μM^{-1} s^{-1}
k_{on} = 6.0 ± 0.3 μM^{-1} s^{-1}
H
PSG:CRIPT$_{P-3}$ 15 AA
PSG:CRIPT 15 AA
k_{on} = 7.0 ± 0.5 μM^{-1} s^{-1}
k_{on} = 8.2 ± 0.3 μM^{-1} s^{-1}
k_{obs}(s^{-1})
[CRIPT 6 AA] or [CRIPT$_{P-3}$ 6 AA] μM
[CRIPT 15 AA] or [CRIPT$_{P-3}$ 15 AA] μM
I
J
K
L
M
N
O
P
k_{off} = 11 ± 0.2 s^{-1}
k_{off} = 2.6 ± 0.06 s^{-1}
k_{off} = 81 ± 0.2 s^{-1}
k_{off} = 12 ± 0.1 s^{-1}
k_{off} = 2.1 ± 0.2 s^{-1}
k_{off} = 1.3 ± 0.08 s^{-1}
k_{off} = 12 ± 0.1 s^{-1}
k_{off} = 3.5 ± 0.03 s^{-1}
[Unlabeled CRIPT 6 AA] μM

Fig. S5.

Binding and dissociation kinetics of CRIPT peptides and PDZ3 or PSG

A-**D** Examples of kinetic traces from stopped flow experiments for binding of dansylated CRIPT (4 µM) CRIPT 6 AA (**A** and **C**) or CRIPT 15 AA (**B** and **D**) to PDZ3 (1 µM) (**A** and **B**) or PSG (1 µM) (**C** or **D**). Traces were fitted to single (**A**, **B** and **D**) or double exponential functions (**C**). **E**-**H** The experiments were repeated over a range of dansylated CRIPT concentrations (2 to 20 µM) at a constant concentration of PDZ3 or PSG. The observed rate constant (k_{obs}) was obtained from the fit to single or double exponential functions and plotted versus [CRIPT]. From the linear relationship between [CRIPT] and k_{obs} the association rate constant (k_{on}) was obtained as the slope at high peptide concentration. **I**-**L** Examples of kinetic traces from stopped flow experiments in which k_{off} was determined in a displacement reaction. A large excess of unlabeled CRIPT was mixed with pre-equilibried protein:peptide complex (2:10 µM) of the respective PDZ3 or PSG and dansyl labeled CRIPT. Traces were fitted to a single (**I**, **J** and **L**) or double (**K**) exponential function to obtain the observed rate constant. Note that the fitting errors of the k_{obs} values are very small (generally less than 0.6 % of the value) due to high signal to noise in combination with 5-6 technical replicates for each kinetic trace **M**-**P** The experiments were repeated with three different concentrations of unlabeled CRIPT (100, 150 and 200 µM) and an average of the observed rate constants was used as the dissociation rate constant (k_{off}). Color codes for protein:peptide complex: pink (PDZ3:CRIPT 6 AA), green (PDZ3: CRIPT 15 AA), blue (PSG:CRIPT 6 AA), light green (PSG:CRIPT 15 AA) and black (the same peptide but with P_{-3} (Nva) mutation). All fitted parameters are presented in Tables S2-S7.

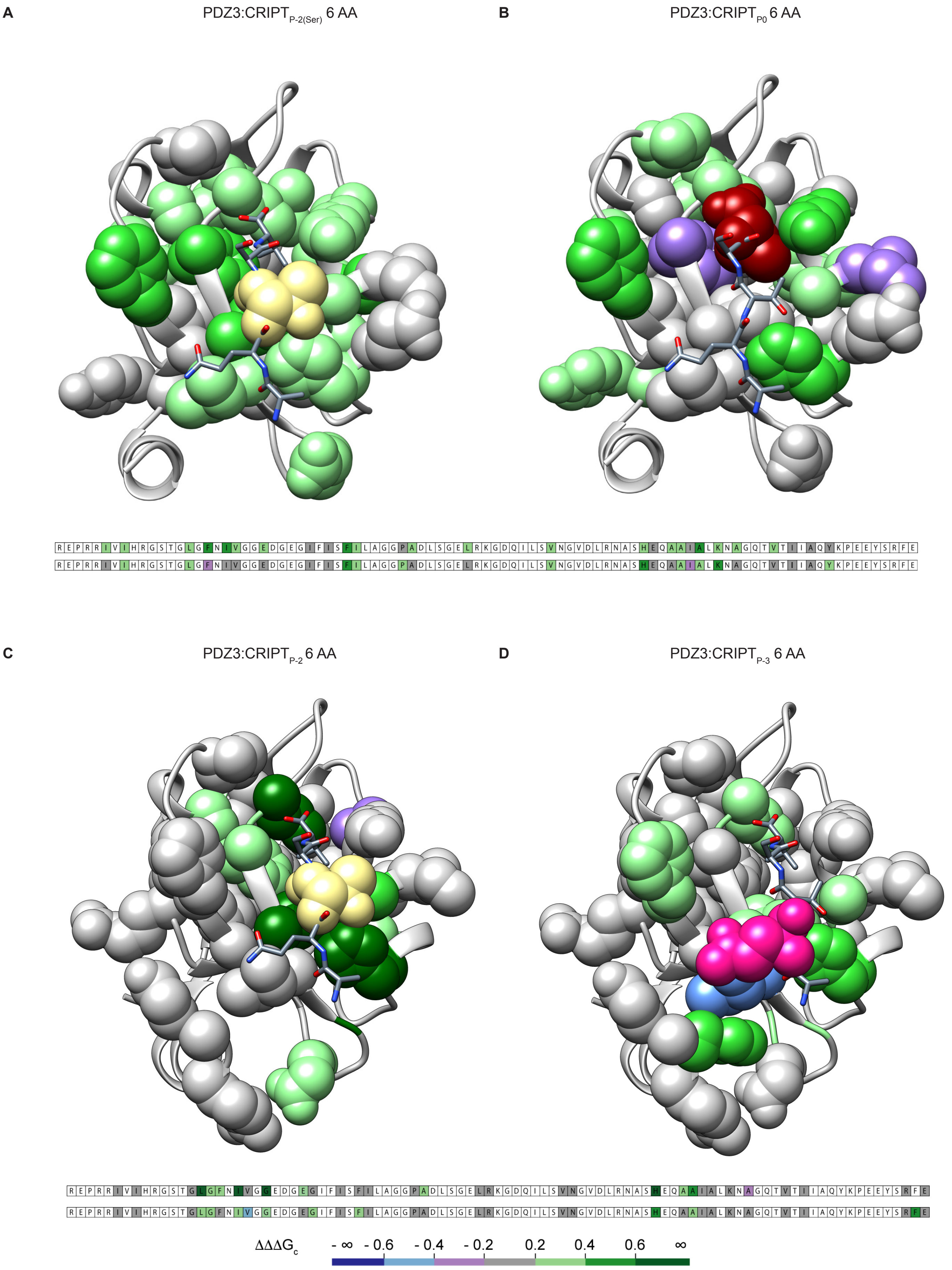

Fig. S6.

Origin of perturbations affects the allosteric network in PDZ3

Comparison of mapped allosteric networks ($\Delta\Delta G_c$) in PDZ3 for four peptide mutations at different positions: **A** P_{-2}: Thr to Ser **B** P_0: Val to Abu **C** P_{-2}: Thr to Abu and **D** P_{-3}: Gln to Nva. The mapped allosteric networks reported in **A** and **B** were published previously [4], whereas those shown in **C** and **D** are from the present work.

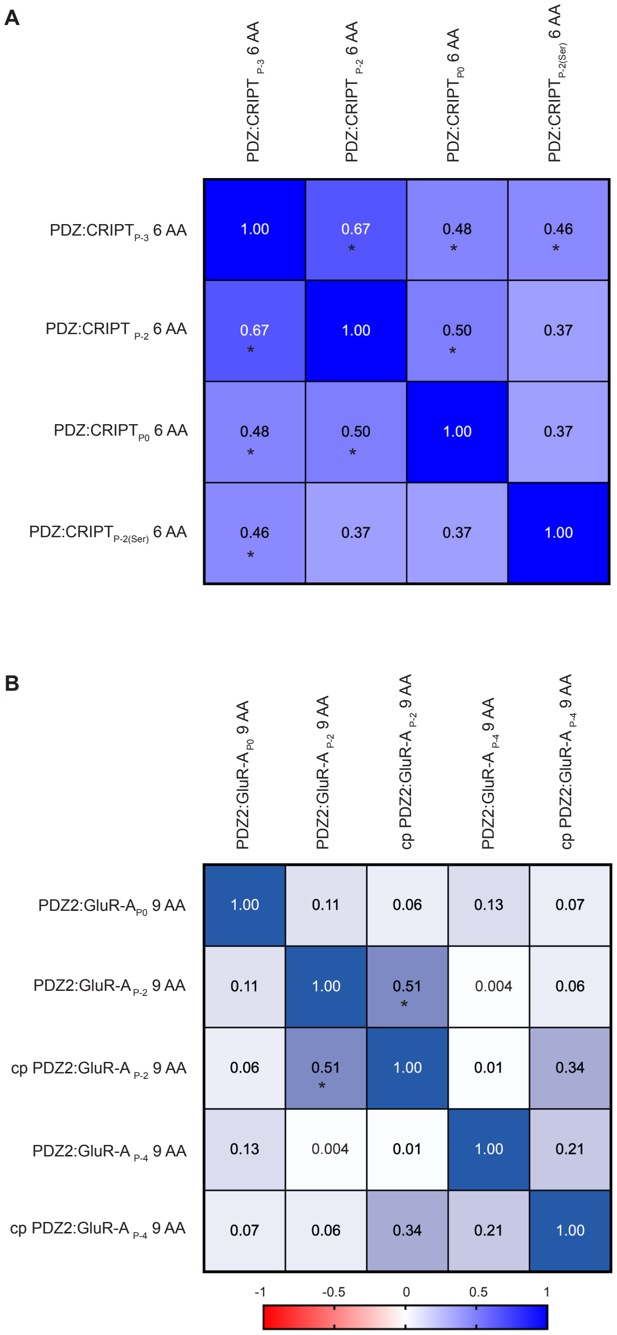

Fig. S7.

Spearman correlation analysis of allosteric networks involving PDZ3

The diagrams report Spearman rank correlation values for allosteric networks mapped by double mutant cycles with two different PDZ domains and mutations in protein and peptide: **A** Four allosteric networks reported in PDZ3 with 22 protein mutations and four peptide mutations: CRIPT$_{P0}$ 6 AA [4], CRIPT$_{P-2}$ 6 AA, CRIPT$_{P-2(Ser)}$ 6 AA [4] and CRIPT$_{P-3}$ 6 AA. **B** Five allosteric networks in SAP97 PDZ2 and a circularly permuted (cp) SAP97 PDZ2 with 19 protein mutations and three peptide mutations: GluR-A$_{P0}$ 9 AA (Val to Abu), GluR-A$_{P-2}$ 9 AA (Thr to Ser) and GluR-A$_{P-4}$ 9 AA (Arg to Nva) [10]. The color code going from dark blue (positive) to red (negative) shows the direction and strength of any monotonic relationship. Significant correlation coefficients are marked with *.

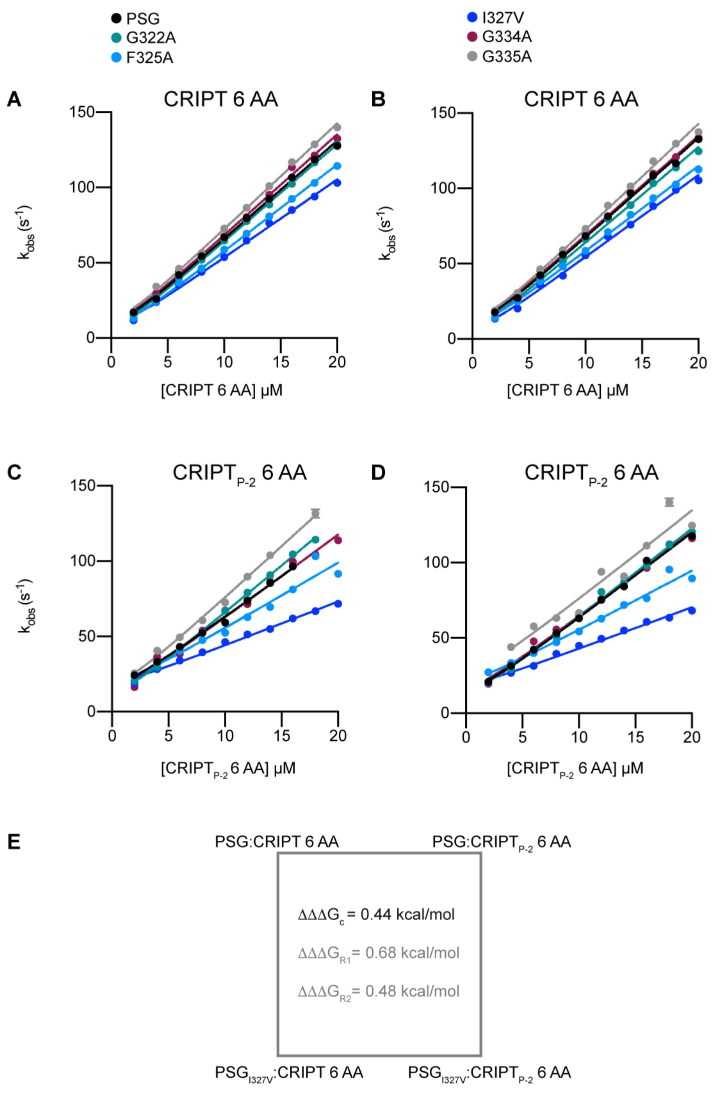

F

	$\Delta\Delta\Delta G_{R1}$	$\Delta\Delta\Delta G_{R2}$	$\Delta\Delta\Delta G_c$
G322A	- 0.69 ± 0.08	- 0.72 ± 0.09	- 0.80 ± 0.11
F325A	0.23 ± 0.11	0.21 ± 0.15	0.42 ± 0.13
I327V	0.68 ± 0.10	0.48 ± 0.11	0.44 ± 0.12
E334A	0.21 ± 0.11	0.07 ± 0.11	0.18 ± 0.12
G335A	- 0.33 ± 0.04	-0.47 ± 0.10	-0.45 ± 0.12

Fig. S8.

Reproducibility of coupling free energies as determined by stopped flow spectroscopy

Five complete double mutant cycles were repeated two times to show the reproducibility of the experimentally determined $\Delta\Delta\Delta G_c$ values. We chose double mutant cycles spanning a range of values from positive to negative. **A** First and **B** second repeat of stopped flow experiments for binding of dansylated CRIPT 6 AA to PSG (1 μM) or the PSG mutants G322A, F325A, I327V, E334A and G335A, respectively. The observed rate constant (k_{obs}) was plotted versus [CRIPT] (2-20 μM). From the linear relationship between [CRIPT] and k_{obs} the association rate constant (k_{on}) was obtained as the slope. **C** First and **D** second repeat of the corresponding stopped flow experiments for binding of dansylated CRIPT$_{P-2}$ 6 AA to PSG and the mutants. For each complex, the dissociation rate constant was determined in displacement experiments. **E** One example of a thermodynamic double mutant cycle for the PSG/peptide mutant pair: I327V/CRIPT$_{P-2}$ 6 AA. Coupling free energies are shown for the repeats ($\Delta\Delta\Delta G_{R1}$ and $\Delta\Delta\Delta G_{R2}$) and the first experiment ($\Delta\Delta\Delta G_c$). **F** Comparison of coupling free energies for the three independent determinations of five different $\Delta\Delta\Delta G_c$

Supplementary Figure References

1. Kaya, C., et al., MCPath: Monte Carlo path generation approach to predict likely allosteric pathways and functional residues. Nucleic Acids Res, 2013. 41(Web Server issue): p. W249-255.
2. Kalescky, R., J. Liu, and P. Tao, Identifying key residues for protein allostery through rigid residue scan. J Phys Chem A, 2015. 119(9): p. 1689-1700.
3. Du, Q.S., et al., Correlation analysis for protein evolutionary family based on amino acid position mutations and application in PDZ domain. PLoS One, 2010. 5(10): p. e13207.
4. Gianni, S., et al., Sequence-specific long range networks in PSD-95/discs large/ZO-1 (PDZ) domains tune their binding selectivity. J Biol Chem, 2011. 286(31): p. 27167-27175.
5. Salinas, V.H. and R. Ranganathan, Coevolution-based inference of amino acid interactions underlying protein function. Elife, 2018. 7.
6. Kumawat, A. and S. Chakrabarty, Hidden electrostatic basis of dynamic allostery in a PDZ domain. Proc Natl Acad Sci U S A, 2017. 114(29): p. E5825-E5834.
7. McLaughlin, R.N., Jr., et al., The spatial architecture of protein function and adaptation. Nat ure, 2012. 491(7422): p. 138-142.
8. Gerek, Z.N. and S.B. Ozkan, Change in allosteric network affects binding affinities of PDZ domains: analysis through perturbation response scanning. PLoS Comput Biol, 2011. 7(10): p. e1002154.
9. Gautier C, Laursen L, Jemth P, Gianni S. Seeking allosteric networks in PDZ domains. Protein Eng Des Sel. 2018;31(10):367-73
10. Hultqvist, G., et al., Energetic pathway sampling in a protein interaction domain. Structure, 2013. 21(7): p. 1193-1202.

Table S1.

Coupling free energies ($\Delta\Delta G_c$) for all double mutant cycles in PDZ3. Color code according to the bar scale.

	$\Delta\Delta G_c^{PDZ3}$	
	CRIPT$_{P-3}$ 6 AA	CRIPT$_{P-2}$ 6 AA
I314V	- 0.02 ± 0.03	- 0.01 ± 0.03
I316A	0.01 ± 0.03	- 0.09 ± 0.03
G322A	0.14 ± 0.04	- 0.04 ± 0.04
L323A	0.20 ± 0.04	0.63 ± 0.10
G324A	0.30 ± 0.05	0.24 ± 0.09
F325A	0.18 ± 0.04	0.29 ± 0.05
I327V	0.21 ± 0.03	0.73 ± 0.08
V328A	- 0.45 ± 0.04	0.10 ± 0.09
G330A	0.33 ± 0.08	1.23 ± 0.08
E334A	0.06 ± 0.03	0.21 ± 0.04
G335A	0.30 ± 0.05	- 0.03 ± 0.05
I338A	- 0.15 ± 0.03	- 0.12 ± 0.03
F340A	0.21 ± 0.03	0.10 ± 0.04
I341V	0.03 ± 0.03	0.04 ± 0.03
P346G	0.02 ± 0.04	0.003 ± 0.059
A347G	0.19 ± 0.04	0.20 ± 0.05
L353A	- 0.06 ± 0.03	- 0.11 ± 0.04
R354A	- 0.06 ± 0.03	- 0.07 ± 0.04
V362A	- 0.03 ± 0.03	0.12 ± 0.03
N363A	0.11 ± 0.04	0.15 ± 0.04
H372A	0.47 ± 0.06	1.53 ± 0.06
A375G	0.02 ± 0.03	0.21 ± 0.04
A376G	0.25 ± 0.03	0.49 ± 0.04
I377A	0.01 ± 0.05	- 0.13 ± 0.03
A378G	- 0.07 ± 0.04	- 0.06 ± 0.03
K380A	0.04 ± 0.03	0.13 ± 0.05
A382G	0.05 ± 0.03	- 0.21 ± 0.03
V386A	- 0.07 ± 0.03	- 0.09 ± 0.03
I388V	- 0.08 ± 0.03	- 0.02 ± 0.03
R399A	- 0.06 ± 0.09	0.02 ± 0.09
F400A	0.57 ± 0.05	N.D.
E401A	- 0.04 ± 0.03	- 0.10 ± 0.05

$\Delta\Delta G_c$ - ∞ - 0.6 - 0.4 - 0.2 0.2 0.4 0.6 ∞

Table S2.

k_{on}, k_{off} and K_d for the interaction between PDZ3 and CRIPT 6 AA or CRIPT $_{P\text{-}3}$ 6 AA.

	CRIPT 6 AA			CRIPT$_{P\text{-}3}$ 6 AA		
	k_{on} (s^{-1} μM^{-1})	k_{off} (s^{-1})	K_d (μM)	k_{on} (s^{-1} μM^{-1})	k_{off} (s^{-1})	K_d (μM)
PDZ3	7.0 ± 0.1	2.6 ± 0.06	0.37 ± 0.02	6.3 ± 0.1	11 ± 0.2	1.7 ± 0.03
I314V	7.1 ± 0.04	2.4 ± 0.04	0.34 ± 0.01	6.6 ± 0.1	11 ± 0.3	1.7 ± 0.1
I316A	6.3 ± 0.03	1.5 ± 0.03	0.24 ± 0.005	5.7 ± 0.1	6 ± 0.1	1.1 ± 0.02
G322A	6.2 ± 0.1	0.7 ± 0.02	0.12 ± 0.004	6.0 ± 0.1	3 ± 0.1	0.42 ± 0.01
L323A	7.1 ± 0.04	1 3 ± 0.3	1.8 ± 0.04	6.5 ± 0.1	37 ± 1	5.7 ± 0.2
G324A	6.7 ± 0.1	12 ± 0.3	1.9 ± 0.1	6.3 ± 0.3	31 ± 2	5.0 ± 0.4
F325A	8.0 ± 0.2	5.8 ± 0.2	0.72 ± 0.03	7.8 ± 0.1	19 ± 0.7	2.5 ± 0.1
I327V	6.1 ± 0.04	14 ± 0.1	2.3 ± 0.02	5.6 ± 0.1	40 ± 0.1	7.2 ± 0.1
V328A	7.1 ± 0.1	7.2 ± 0.2	1.0 ± 0.03	7.5 ± 0.1	79 ± 2	10.6 ± 0.3
G330A	6.3 ± 0.2	65 ± 3	10 ± 0.6	8.0 ± 0.8	216 ± 18	27 ± 3
E334A	9.2 ± 0.1	5.6 ± 0.1	0.61 ± 0.01	7.6 ± 0.1	20 ± 0.04	2.6 ± 0.04
G335A	14 ± 0.2	0.5 ± 0.03	0.038 ± 0.002	13 ± 0.2	1 ± 0.1	0.10 ± 0.005
I338A	6.0 ± 0.03	1.9 ± 0.04	0.31 ± 0.01	5.4 ± 0.0	10 ± 0.2	1.9 ± 0.1
F340A	6.3 ± 0.1	4.4 ± 0.1	0.70 ± 0.01	5.8 ± 0.1	13 ± 0.5	2.3 ± 0.1
I341V	7.0 ± 0.04	3.4 ± 0.1	0.49 ± 0.01	6.4 ± 0.1	14 ± 0.3	2.2 ± 0.1
P346G	6.8 ± 0.1	4.1 ± 0.2	0.60 ± 0.03	6.1 ± 0.1	17 ± 0.6	2.7 ± 0.1
A347G	5.8 ± 0.03	5.8 ± 0.3	1.0 ± 0.1	5.2 ± 0.1	17 ± 0.2	3.3 ± 0.1
L353A	6.7 ± 0.03	1.9 ± 0.1	0.29 ± 0.01	6.0 ± 0.1	9 ± 0.1	1.5 ± 0.03
R354A	7.9 ± 0.1	2.7 ± 0.04	0.34 ± 0.01	6.2 ± 0.1	11 ± 0.1	1.8 ± 0.03
V362A	5.6 ± 0.1	2.4 ± 0.02	0.42 ± 0.01	5.2 ± 0.1	11 ± 0.1	2.1 ± 0.04
N363A	6.8 ± 0.1	4.0 ± 0.1	0.60 ± 0.02	6.2 ± 0.1	14 ± 0.3	2.3 ± 0.1
H372A	5.3 ± 0.1	35 ± 0.4	6.5 ± 0.1	9.9 ± 0.7	131 ± 9	13 ± 1
A375G	5.1 ± 0.1	6.1 ± 0.1	1.2 ± 0.03	4.6 ± 0.1	25 ± 0.1	5.4 ± 0.1
A376G	7.2 ± 0.03	10 ± 0.4	1.4 ± 0.1	6.4 ± 0.1	28 ± 0.1	4.3 ± 0.1
I377A	6.9 ± 0.04	2.1 ± 0.01	0.31 ± 0.002	6.1 ± 0.1	9 ± 0.6	1.4 ± 0.1
A378G	6.5 ± 0.1	1.5 ± 0.01	0.23 ± 0.002	6.5 ± 0.1	8 ± 0.3	1.2 ± 0.1
K380A	5.4 ± 0.03	5.1 ± 0.1	0.94 ± 0.02	5.0 ± 0.1	21 ± 0.2	4.1 ± 0.1
A382G	6.9 ± 0.04	1.0 ± 0.01	0.14 ± 0.001	6.3 ± 0.1	4 ± 0.1	0.62 ± 0.01
V386A	6.6 ± 0.04	1.9 ± 0.1	0.29 ± 0.01	6.0 ± 0.0	9 ± 0.1	1.5 ± 0.02
I388V	7.2 ± 0.1	3.3 ± 0.04	0.45 ± 0.01	6.4 ± 0.1	16 ± 0.3	2.4 ± 0.1
R399A	8.0 ± 0.1	7.3 ± 0.1	0.91 ± 0.01	5.8 ± 0.1	27 ± 0.1	4.7 ± 0.1
F400A	7.5 ± 0.5	110 ± 4	15 ± 1	9.1 ± 4	229 ± 49	25 ± 11
E401A	7.3 ± 0.1	5.3 ± 0.03	0.72 ± 0.01	6.2 ± 0.2	22 ± 0.1	3.6 ± 0.1

Table S3.

k_{on}, k_{off} and K_d for the interaction between PDZ3 and CRIPT 6 AA or CRIPT $_{P-2}$ 6 AA.

	CRIPT 6 AA			CRIPT$_{P-2}$ 6 AA		
	k_{on} (s^{-1} μM^{-1})	k_{off} (s^{-1})	K_d (μM)	k_{on} (s^{-1} μM^{-1})	k_{off} (s^{-1})	K_d (μM)
PDZ3	7.0 ± 0.1	2.6 ± 0.06	0.37 ± 0.02	7.5 ± 0.1	68 ± 0.2	9.0 ± 0.2
I314V	7.1 ± 0.04	2.4 ± 0.04	0.34 ± 0.01	7.6 ± 0.2	64 ± 0.6	8.5 ± 0.2
I316A	6.3 ± 0.03	1.5 ± 0.03	0.24 ± 0.005	6.3 ± 0.1	43 ± 0.3	6.8 ± 0.1
G322A	6.2 ± 0.1	0.7 ± 0.02	0.12 ± 0.004	6.2 ± 0.1	19 ± 0.2	3.0 ± 0.1
L323A	7.1 ± 0.04	1 3 ± 0.3	1.8 ± 0.04	16 ± 2	221 ± 26	14 ± 2
G324A	6.7 ± 0.1	12 ± 0.3	1.9 ± 0.1	6.6 ± 0.7	193 ± 17	29 ± 4
F325A	8.0 ± 0.2	5.8 ± 0.2	0.72 ± 0.03	9.0 ± 0.4	94 ± 5	11 ± 0.7
I327V	6.1 ± 0.04	14 ± 0.1	2.3 ± 0.02	12 ± 1	172 ± 15	15 ± 2
V328A	7.1 ± 0.1	7.2 ± 0.2	1.0 ± 0.03	8.5 ± 1	175 ± 9	21 ± 3
G330A	6.3 ± 0.2	65 ± 3	10 ± 0.6	7.3 ± 0.7	203 ± 16	28 ± 3
E334A	9.2 ± 0.1	5.6 ± 0.1	0.61 ± 0.01	9.8 ± 0.3	99 ± 4	10 ± 0.5
G335A	14 ± 0.2	0.5 ± 0.03	0.038 ± 0.002	15 ± 0.3	14 ± 0.1	1.0 ± 0.02
I338A	6.0 ± 0.03	1.9 ± 0.04	0.31 ± 0.01	6.1 ± 0.1	57 ± 0.3	9.3 ± 0.2
F340A	6.3 ± 0.1	4.4 ± 0.1	0.70 ± 0.01	6.7 ± 0.2	95 ± 1	14 ± 0.5
I341V	7.0 ± 0.04	3.4 ± 0.1	0.49 ± 0.01	8.2 ± 0.2	90 ± 0.4	11 ± 0.3
P346G	6.8 ± 0.1	4.1 ± 0.2	0.60 ± 0.03	6.7 ± 0.5	97 ± 1	15 ± 1
A347G	5.8 ± 0.03	5.8 ± 0.3	1.0 ± 0.1	7.9 ± 0.4	133 ± 5	17 ± 1
L353A	6.7 ± 0.03	1.9 ± 0.1	0.29 ± 0.01	6.9 ± 0.2	57 ± 0.7	8.4 ± 0.2
R354A	7.9 ± 0.1	2.7 ± 0.04	0.34 ± 0.01	8.0 ± 0.3	75 ± 2	9.3 ± 0.4
V362A	5.6 ± 0.1	2.4 ± 0.02	0.42 ± 0.01	5.7 ± 0.1	47 ± 0.7	8.2 ± 0.2
N363A	6.8 ± 0.1	4.0 ± 0.1	0.60 ± 0.02	8.1 ± 0.3	89 ± 2	11 ± 0.6
H372A	5.3 ± 0.1	35 ± 0.4	6.5 ± 0.1	7.5 ± 0.5	78 ± 6	10 ± 1
A375G	5.1 ± 0.1	6.1 ± 0.1	1.2 ± 0.03	6.2 ± 0.2	123 ± 5	20 ± 1
A376G	7.2 ± 0.03	10 ± 0.4	1.4 ± 0.1	8.2 ± 0.3	121 ± 4	15 ± 0.7
I377A	6.9 ± 0.04	2.1 ± 0.01	0.31 ± 0.002	7.3 ± 0.1	68 ± 0.5	9.3 ± 0.2
A378G	6.5 ± 0.1	1.5 ± 0.01	0.23 ± 0.002	7.2 ± 0.1	45 ± 0.8	6.2 ± 0.1
K380A	5.4 ± 0.03	5.1 ± 0.1	0.94 ± 0.02	6.3 ± 0.4	112 ± 4	18 ± 1
A382G	6.9 ± 0.04	1.0 ± 0.01	0.14 ± 0.001	6.9 ± 0.1	35 ± 0.1	5.0 ± 0.1
V386A	6.6 ± 0.04	1.9 ± 0.1	0.29 ± 0.01	6.5 ± 0.1	53 ± 0.3	8.2 ± 0.2
I388V	7.2 ± 0.1	3.3 ± 0.04	0.45 ± 0.01	7.5 ± 0.2	85 ± 0.8	11 ± 0.3
R399A	8.0 ± 0.1	7.3 ± 0.1	0.91 ± 0.01	9.2 ± 1	193 ± 14	21 ± 3
F400A	7.5 ± 0.5	110 ± 4	15 ± 1			
E401A	7.3 ± 0.1	5.3 ± 0.03	0.72 ± 0.01	7.8 ± 0.5	163 ± 6	21 ± 2

Table S4.

k_{on}, k_{off} and K_d for the interaction between PSG and CRIPT 6 AA or CRIPT $_{P\text{-}3}$ 6 AA.

	CRIPT 6 AA			CRIPT$_{P\text{-}3}$ 6 AA		
	k_{on} (s^{-1} μM^{-1})	k_{off} (s^{-1})	K_d (μM)	k_{on} (s^{-1} μM^{-1})	k_{off} (s^{-1})	K_d (μM)
PSG	6.0 ± 0.3	1.3 ± 0.1	0.21 ± 0.01	5.8 ± 0.2	2.1 ± 0.2	0.36 ± 0.02
G322A	6.8 ± 0.1	0.26 ± 0.002	0.039 ± 0.001	6.0 ± 0.1	1.0 ± 0.1	0.16 ± 0.01
F325A	5.5 ± 0.2	4.7 ± 0.5	0.87 ± 0.1	5.2 ± 0.3	8.3 ± 0.3	1.6 ± 0.1
I327V	5.7 ± 0.1	8.9 ± 1.0	1.6 ± 0.2	4.4 ± 0.2	15 ± 0.1	3.4 ± 0.1
G330A	6.2 ± 0.2	35 ± 1.8	5.8 ± 0.3	6.6 ± 0.2	85 ± 2.6	13 ± 0.6
E334A	7.2 ± 0.1	2.4 ± 0.1	0.33 ± 0.01	6.4 ± 0.2	6.8 ± 0.1	1.1 ± 0.04
G335A	7.0 ± 0.1	0.74 ± 0.03	0.11 ± 0.01	6.9 ± 0.2	1.8 ± 0.03	0.26 ± 0.01
I338A	5.4 ± 0.1	1.1 ± 0.03	0.20 ± 0.01	4.7 ± 0.1	2.7 ± 0.2	0.58 ± 0.04
A347G	4.4 ± 0.1	5.0 ± 0.3	1.1 ± 0.1	3.8 ± 0.1	14 ± 0.6	3.6 ± 0.2
L353A	6.3 ± 0.1	1.1 ± 0.1	0.18 ± 0.01	5.6 ± 0.1	3.3 ± 0.2	0.59 ± 0.03
R354A	7.0 ± 0.1	1.7 ± 0.1	0.25 ± 0.02	5.9 ± 0.1	4.7 ± 0.1	0.80 ± 0.03
V362A	7.1 ± 0.4	1.3 ± 0.01	0.19 ± 0.01	5.4 ± 0.3	6.8 ± 0.9	1.3 ± 0.2
A376G	6.6 ± 0.1	5.4 ± 0.2	0.82 ± 0.03	5.6 ± 0.2	14 ± 1.2	2.4 ± 0.2
R399A	7.2 ± 0.2	2.8 ± 0.3	0.38 ± 0.04	6.3 ± 0.2	6.7 ± 0.4	1.1 ± 0.1
F400A	6.4 ± 0.3	13 ± 0.4	2.0 ± 0.1	4.0 ± 0.1	29 ± 0.4	7.2 ± 0.2
E401A	5.6 ± 0.1	5.1 ± 0.5	0.90 ± 0.1	5.1 ± 0.3	7.3 ± 0.4	1.4 ± 0.1

Table S5.

k_{on}, k_{off} and K_d for the interaction between PSG and CRIPT 6 AA or CRIPT $_{P-2}$ 6 AA

	CRIPT 6 AA			CRIPT$_{P-2}$ 6 AA		
	k_{on} (s^{-1} μM^{-1})	k_{off} (s^{-1})	K_d (μM)	k_{on} (s^{-1} μM^{-1})	k_{off} (s^{-1})	K_d (μM)
PSG	6.0 ± 0.3	1.3 ± 0.1	0.21 ± 0.01	5.2 ± 0.002	10 ± 2	2.0 ± 0.4
G322A	6.8 ± 0.1	0.26 ± 0.002	0.039 ± 0.001	5.9 ± 0.1	9.1 ± 0.6	1.5 ± 0.1
F325A	5.5 ± 0.2	4.7 ± 0.5	0.87 ± 0.1	5.4 ± 0.4	21 ± 0.4	3.9 ± 0.3
I327V	5.7 ± 0.1	8.9 ± 1	1.6 ± 0.2	3.1 ± 0.1	21 ± 0.5	6.8 ± 0.2
G330A	6.2 ± 0.2	35 ± 2	5.8 ± 0.3	6.4 ± 0.5	142 ± 7	22 ± 2
E334A	7.2 ± 0.1	2.4 ± 0.1	0.33 ± 0.01	7.4 ± 0.3	17 ± 1	2.3 ± 0.2
G335A	7.0 ± 0.1	0.74 ± 0.03	0.11 ± 0.01	7.5 ± 0.2	17 ± 1	2.2 ± 0.2
I338A	5.4 ± 0.1	1.1 ± 0.03	0.20 ± 0.01	4.1 ± 0.1	17 ± 1.0	4.1 ± 0.3
A347G	4.4 ± 0.1	5.0 ± 0.3	1.1 ± 0.1	3.7 ± 0.2	31 ± 0.01	8.4 ± 0.4
L353A	6.3 ± 0.1	1.1 ± 0.1	0.18 ± 0.01	6.2 ± 0.3	16 ± 0.9	2.6 ± 0.2
R354A	7.0 ± 0.1	1.7 ± 0.1	0.25 ± 0.02	5.2 ± 0.2	17 ± 0.4	3.3 ± 0.2
V362A	7.1 ± 0.4	1.3 ± 0.01	0.19 ± 0.01	5.0 ± 0.8	20 ± 1	4.1 ± 0.7
A376G	6.6 ± 0.1	5.4 ± 0.2	0.82 ± 0.03	4.5 ± 0.1	26 ± 0.3	5.8 ± 0.1
R399A	7.2 ± 0.2	2.8 ± 0.3	0.38 ± 0.04	8.6 ± 0.6	71 ± 4	8.2 ± 0.7
F400A	6.4 ± 0.3	13 ± 0.4	2.0 ± 0.1			
E401A	5.6 ± 0.1	5.1 ± 0.5	0.90 ± 0.1	5.1 ± 1	64 ± 4	13 ± 3

Table S6.

k_{on}, k_{off} and K_d for the interaction between PDZ3 and CRIPT 15 AA or CRIPT $_{P-3}$ 15 AA.

	CRIPT 15 AA			CRIPT$_{P-3}$ 15 AA		
	k_{on} (s^{-1} μM^{-1})	k_{off} (s^{-1})	K_d (μM)	k_{on} (s^{-1} μM^{-1})	k_{off} (s^{-1})	K_d (μM)
PDZ3	8.6 ± 0.1	12 ± 0.1	1.4 ± 0.02	8.4 ± 0.2	81 ± 0.2	9.7 ± 0.3
G322A	8.0 ± 0.1	3.5 ± 0.02	0.44 ± 0.01	7.6 ± 0.1	20 ± 0.1	2.7 ± 0.03
F325A	11 ± 0.2	26 ± 0.2	2.4 ± 0.04	11 ± 0.6	125 ± 6	11 ± 0.8
I327V	7.6 ± 0.1	62 ± 1	8.2 ± 0.2	6.1 ± 0.7	397 ± 11	65 ± 8
G330A	8.7 ± 0.3	97 ± 0.4	11 ± 0.4	8.1 ± 3.4	307 ± 37	38 ± 17
E334A	8.3 ± 0.1	22 ± 0.2	2.6 ± 0.1	10 ± 0.6	130 ± 7	13 ± 1
G335A	12 ± 0.2	4.5 ± 0.01	0.36 ± 0.01	11 ± 0.2	24 ± 0.3	2.1 ± 0.05
I338A	7.2 ± 0.1	11 ± 0.1	1.5 ± 0.03	5.6 ± 0.2	65 ± 2	12 ± 0.4
A347G	7.3 ± 0.2	25 ± 0.2	3.4 ± 0.1	5.8 ± 0.3	135 ± 4	23 ± 1
L353A	8.1 ± 0.1	12 ± 0.03	1.5 ± 0.03	5.8 ± 0.3	85 ± 1	15 ± 0.8
R354A	9.1 ± 0.1	12 ± 0.1	1.4 ± 0.04	9.4 ± 0.3	87 ± 2	9.2 ± 0.3
V362A	7.3 ± 0.1	9.8 ± 0.04	1.3 ± 0.03	8.3 ± 0.2	72 ± 2	8.7 ± 0.3
A376G	8.6 ± 0.2	39 ± 0.5	4.5 ± 0.1	11 ± 0.7	190 ± 8	17 ± 1
R399A	11 ± 0.1	7.8 ± 0.1	0.70 ± 0.01	11 ± 0.1	38 ± 0.4	3.4 ± 0.05
F400A	9.8 ± 0.1	13 ± 0.1	1.3 ± 0.02	15 ± 0.4	72 ± 1	4.9 ± 0.2
E401A	7.5 ± 0.1	15 ± 0.1	2.0 ± 0.04	7.8 ± 0.2	96 ± 0.6	12 ± 0.4

Table S7.
k_{on}, k_{off} and K_d for the interaction between PSG and CRIPT 15 AA or CRIPT $_{P\text{-}3}$ 15 AA.

	CRIPT 15 AA			CRIPT$_{P\text{-}3}$ 15 AA		
	k_{on} (s^{-1} μM^{-1})	k_{off} (s^{-1})	K_d (μM)	k_{on} (s^{-1} μM^{-1})	k_{off} (s^{-1})	K_d (μM)
PSG	8.2 ± 0.3	3.5 ± 0.03	0.43 ± 0.01	7.0 ± 0.5	12 ± 0.1	1.7 ± 0.2
G322A	8.2 ± 0.1	0.8 ± 0.01	0.10 ± 0.002	7.0 ± 0.1	2.4 ± 0.03	0.34 ± 0.01
F325A	6.2 ± 0.2	8.8 ± 0.1	1.41 ± 0.05	6.1 ± 0.3	22 ± 0.2	3.6 ± 0.2
I327V	6.3 ± 0.2	19 ± 0.6	3.0 ± 0.1	5.6 ± 0.3	52 ± 0.1	9.2 ± 0.5
G330A	7.7 ± 0.3	99 ± 2.6	13 ± 0.7	15 ± 2	208 ± 13	14 ± 2
E334A	9.4 ± 0.1	8.4 ± 0.2	0.90 ± 0.02	8.2 ± 0.3	28 ± 0.5	3.4 ± 0.1
G335A	8.3 ± 0.1	1.8 ± 0.03	0.21 ± 0.005	7.2 ± 0.1	5.3 ± 0.1	0.73 ± 0.02
I338A	7.1 ± 0.1	2.7 ± 0.01	0.38 ± 0.004	6.2 ± 0.1	8.2 ± 0.04	1.3 ± 0.02
A347G	4.8 ± 0.1	8.9 ± 0.4	1.9 ± 0.1	2.9 ± 0.2	35 ± 0.7	12 ± 1
L353A	8.7 ± 0.1	2.8 ± 0.01	0.32 ± 0.005	7.4 ± 0.2	8.9 ± 0.1	1.2 ± 0.03
R354A	8.1 ± 0.2	3.9 ± 0.03	0.48 ± 0.01	7.8 ± 0.2	13 ± 0.2	1.6 ± 0.04
V362A	4.7 ± 0.3	3.6 ± 0.2	0.78 ± 0.1	6.5 ± 0.1	12 ± 0.1	1.9 ± 0.03
A376G	8.1 ± 0.1	12 ± 0.1	1.4 ± 0.02	5.4 ± 0.2	28 ± 0.3	5.1 ± 0.2
R399A	8.1 ± 0.2	5.0 ± 0.1	0.62 ± 0.02	8.0 ± 0.1	15 ± 0.1	1.8 ± 0.03
F400A	6.3 ± 0.1	6.4 ± 0.1	1.0 ± 0.02	6.9 ± 0.3	41 ± 1	6.0 ± 0.3
E401A	6.8 ± 0.1	3.4 ± 0.04	0.50 ± 0.01	6.4 ± 0.1	12 ± 0.1	1.9 ± 0.04

Table S8.

Difference in coupling free energy ($\Delta\Delta\Delta G_c^{PSG}$-$\Delta\Delta\Delta G_c^{PDZ3}$ = $\Delta\Delta\Delta\Delta G_c^{PSG\ to\ PDZ3}$) between PSG and PDZ3 for corresponding double mutant cycles involving $CRIPT_{P-2}$ 6 AA, $CRIPT_{P-3}$ 6 AA and $CRIPT_{P-3}$ 15 AA. Color code according to the bar scale. N.D., not determined as $\Delta\Delta\Delta G_c^{F400A}$ not possible to determine for $CRIPT_{P-2}$ 6 AA.

	$CRIPT_{P-2}$ 6 AA $\Delta\Delta\Delta\Delta G_c^{PSG\ to\ PDZ3}$	$CRIPT_{P-3}$ 6 AA $\Delta\Delta\Delta\Delta G_c^{PSG\ to\ PDZ3}$	$CRIPT_{P-3}$ 15 AA $\Delta\Delta\Delta\Delta G_c^{PSG\ to\ PDZ3}$
G322A	- 0.76 ± 0.12	- 0.65 ± 0.07	0.008 ± 0.06
F325A	0.14 ± 0.14	- 0.23 ± 0.09	0.04 ± 0.08
I327V	- 0.29 ± 0.15	- 0.37± 0.08	0.23 ± 0.09
G330A	- 0.73 ± 0.15	- 0.50 ± 0.10	0.34 ± 0.26
E334A	- 0.03 ± 0.13	- 0.43 ± 0.06	- 0.17 ± 0.07
G335A	- 0.42 ± 0.12	- 0.52 ± 0.07	0.01 ± 0.06
I338A	- 0.33 ± 0.12	- 0.17 ± 0.07	0.18 ± 0.06
A347G	- 0.07 ± 0.13	- 0.56 ± 0.07	- 0.26 ± 0.08
L353A	- 0.13 ± 0.13	- 0.32 ± 0.07	0.24 ± 0.06
R354A	- 0.13 ± 0.12	- 0.31 ± 0.07	0.10 ± 0.06
V362A	- 0.60 ± 0.15	- 0.77 ± 0.10	0.27 ± 0.07
A376G	- 0.32 ± 0.12	- 0.58 ± 0.08	- 0.28 ± 0.07
R399A	- 0.49 ± 0.16	- 0.23 ± 0.08	- 0.03 ± 0.06
F400A	N.D.	-1.00 ± 0.07	- 0.54 ± 0.07
E401A	- 0.12 ± 0.19	0.07 ± 0.09	- 0.02 ± 0.06

$\Delta\Delta\Delta\Delta G_c$ - ∞   - 0.6   - 0.4   - 0.2   0.2   0.4   0.6   ∞
